# TBC1d24-ephrinB2 interaction regulates contact inhibition of locomotion in neural crest cell migration

**DOI:** 10.1038/s41467-018-05924-9

**Published:** 2018-08-28

**Authors:** Jaeho Yoon, Yoo-Seok Hwang, Moonsup Lee, Jian Sun, Hee Jun Cho, Laura Knapik, Ira O. Daar

**Affiliations:** 1 National Cancer Institute, Frederick, MD 21702 USA; 2 Department of Biochemistry, Institute of Cell Differentiation and Aging, College of Medicine, Hallym University, ChunCheon, 200702 Kangwon-Do Republic of Korea; 3 Immunotherapy Convergence Research Center, Korea Research Institute of Bioscience and Biotechnology (KRIBB), Daejeon, 34141 Korea

## Abstract

Although Eph-ephrin signalling has been implicated in the migration of cranial neural crest (CNC) cells, it is still unclear how ephrinB transduces signals regulating this event. We provide evidence that TBC1d24, a putative Rab35-GTPase activating protein (Rab35 GAP), complexes with ephrinB2 via the scaffold Dishevelled (Dsh) and mediates a signal affecting contact inhibition of locomotion (CIL) in CNC cells. Moreover, we found that, in migrating CNC, the interaction between ephrinB2 and TBC1d24 negatively regulates E-cadherin recycling in these cells via Rab35. Upon engagement of the cognate Eph receptor, ephrinB2 is tyrosine phosphorylated, which disrupts the ephrinB2/Dsh/TBC1d24 complex. The dissolution of this complex leads to increasing E-cadherin levels at the plasma membrane, resulting in loss of CIL and disrupted CNC migration. Our results indicate that TBC1d24 is a critical player in ephrinB2 control of CNC cell migration via CIL.

## Abstract (web summary)

Group migration of cranial neural crest cells depends upon Eph-ephrin signalling. Here, the authors show that TBC1d24 complexes with ephrinB2 via Dishevelled to induce contact inhibition of locomotion by reducing membrane E-cadherin. Eph receptor binding dissolves this complex and disrupts migration.

## Introduction

Cranial neural crest (CNC) cells arise from neuroectoderm in the early neurula embryo and they undergo collective cell migration after segregation from the ectoderm through at least a partial epithelial-to-mesenchymal transition^1^. Various factors are known to participate in neural crest cell migration. Separation from ectoderm involves a coordinated alteration in the levels of E-cadherin and N-cadherin, as well as cadherin-11^1–9^. The chemotactic response between CNC and placodes that secrete attractant molecules such as stromal cell-derived factor 1 (SDF-1) affects the migratory direction^4^. Other secreted factors, including C3a, semaphorins, glial-derived growth factor, fibroblast growth factors (FGFs) and vascular endothelial growth factors, also play a role^10–17^. Directionality of CNC cell migration also relies upon the non-canonical Wnt/planar cell polarity (PCP) signalling pathway^18,19^, which even influences mechanical cues from the underlying mesoderm tissue to regulate CNC migration^20^. Additionally, CNC cell migration is critically dependent on cell-to-cell interactions. Recently, several groups have shown that E-cadherin levels are downregulated to initiate CNC migration, but a low level of E-cadherin is maintained for migrating CNC cells to regulate cell-to-cell adhesion and motility^1,3,21^.

Eph/ephrin signalling is involved in a number of embryonic developmental processes by regulating cell–cell interaction events. Several studies using the mouse, chick and *Xenopus* systems demonstrate that CNC cells express various combinations of ephrin ligands and Eph receptors to guide directional migration. Loss-of-function studies targeting Eph–ephrin signalling demonstrate that complementary expression of ephrin ligands and Eph receptors generates bi-directional signalling to modulate repulsion or attraction of migratory CNC cells^22–30^. However, it is still unclear how ephrinB mechanistically transduces the signals affecting this repulsion or attraction.

Here we provide evidence that TBC1d24 interacts with ephrinB2. TBC1d24 is a Rab-GAP that has two conserved domains consisting of a TBC (Tre2–Bub2–Cdc16) domain and TLD (TBC LysM) domain, which are predicted to regulate endocytosis and exocytosis of cellular vesicles^31^. In human patients, several mutations in TBC1d24 have been identified, and heterozygous missense mutations have been determined to cause neurological disorders, including DOORS (deafness, onychodystrophy, osteodystrophy, mental retardation and seizures) and familial infantile myoclonic epilepsy^32,33^. In addition, patients with homozygous TBC1d24 truncation mutations display severe neurodegeneration^34^. In our study, loss of TBC1d24 function causes CNC cell migration defects through disruption of CIL, and these defects can be rescued by re-expressing the wild-type protein. However, TBC1d24 interaction mutants that are not able to associate with either ephrinB2 or Rab35 fail to rescue the TBC1d24 loss-of-function phenotype. We show that the ephrinB2 and TBC1d24 interaction modulates contact inhibition of locomotion (CIL) through regulating E-cadherin recycling. Our results provide the molecular mechanism of how ephrinB2 regulates the CIL response during CNC cell migration.

## Results

### The newly identified ephrinB2-binding partner, TBC1d24

**Fig. 1:**
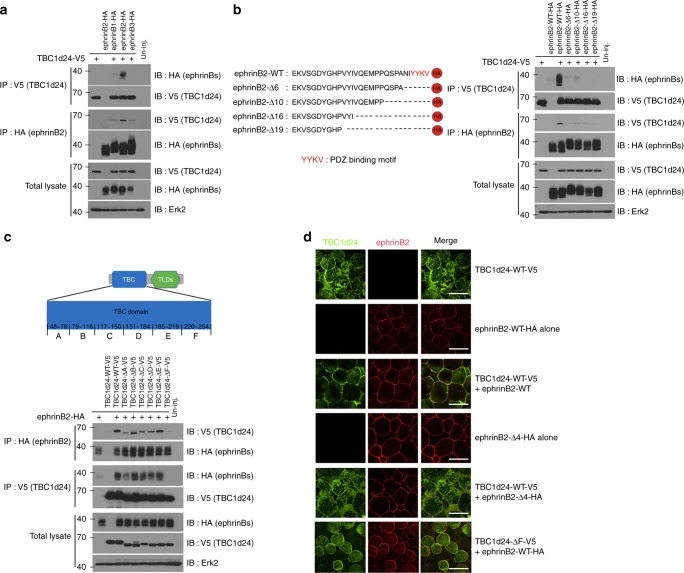
TBC1d24 interacts with ephrinB2. **a** Co-immunoprecipitation assay (Co-IP) using gastrula embryos (stage 10) injected with TBC1d24-V5 RNA (500 pg) and ephrinB1-HA (300 pg), ephrinB2-HA (500 pg) or ephrinB3-HA RNAs (300 pg) show that TBC1d24 interacts with ephrinB2. **b** Illustration of serial deletion mutants from the C-terminus of ephrinB2. Co-IP using gastrula embryos injected with RNA encoding TBC1d24-V5 and HA-tagged ephrinB2 serial deletion mutants shows that the PDZ binding motif (Δ6) of ephrinB2 is required for TBC1d24 interaction. **c** Illustration of the amino acid stretches representing regions deleted within the TBC domain. Co-IP using gastrulas injected with RNA for ephrinB2-HA and TBC1d24-V5 serial deletion mutants shows that the F region within the TBC domain of TBC1d24 is critical for an ephrinB2 interaction. **d** Immunofluorescence microscopic analysis (IF) shows that membrane localisation of V5-tagged TBC1d24 was induced by wild-type ephrinB2 but not ephrinB2-Δ4. Animal caps were dissected at stage 10 and then immunostained for ephrinB2 (red) and TBC1d24 (green). Bar, 50 μm

Several ephrinB-interacting proteins have been discovered that function in pathways regulating cell adhesion and migration (RGS3-PDZ, FGF receptor (FGFR), Dishevelled, Grb4 and CNK1)^35–37^. To identify additional proteins that might mediate ephrinB signalling, we used mass spectrometric analysis of proteins that co-immunoprecipitate (Co-IP) with ephrinB2 when it is overexpressed in *Xenopus* embryos^38^. From this analysis, we identified TBC1d24 as a candidate ephrinB2-interacting protein. Confirmation of a possible interaction between these proteins was provided by Co-IP analysis of lysates from *Xenopus* embryos exogenously expressing tagged versions of TBC1d24 and ephrinB2 (Fig. 1a). Of the three transmembrane ephrinB ligands (B1, B2, B3), ephrinB2 has the most robust interaction with TBC1d24 in Co-IP analyses (Fig. 1a). IP analysis of ephrinB2 from lysates of LS174T human colon carcinoma cells showed that TBC1d24 was found in the ephrinB2 immune-complexes (Supplementary Fig. 1a), indicating that an endogenous interaction exists between ephrinB2 and TBC1d24. Having established that ephrinB2 can associate with TBC1d24, we expressed TBC1d24 in the embryos along with ephrinB2 deletion mutants and found that deletion of six amino acids in the C-terminus encompassing the PDZ-binding motif markedly reduced the interaction with TBC1d24 (Fig. 1b).

In order to find the region within TBC1d24 necessary for an association with ephrinB2, deletion mutants within TBC1d24 were constructed. IP analysis with deletion mutants generated within TBC1d24 (Supplementary Fig. 1b) indicated that the TBC domain was critical for the association between ephrinB2 and TBC1d24 (Supplementary Fig. 1b). Using serial deletion mutants within the TBC domain, it was determined that the 35 amino acids at the 3′ end of the TBC domain (termed the F region) is required for the interaction (Fig. 1c). To address whether the ephrinB2/TBC1d24 interaction affects their localisation, tagged versions of both proteins were expressed alone or along with either a wild-type or interaction mutant version of each protein. In animal pole explants (ectoderm), TBC1d24 alone localised to the cytoplasm; however, ephrinB2 co-expression promotes membrane localisation of TBC1d24. In contrast, TBC1d24 remained in the cytoplasm when co-expressed with the ephrinB2 mutant that lacks the PDZ-binding motif (Fig. 1d). A complementary experiment expressing a TBC1d24 mutant lacking the F region (TBC1d24-ΔF) necessary for an association with ephrinB2 (Fig. 1d) revealed that the TBC1d24-ΔF mutant protein remained in the cytoplasm when co-expressed with the ephrinB2 (Fig. 1d). These data indicate that the association between ephrinB2 and TBC1d24 causes the re-localisation of TBC1d24 from the cytoplasm to the plasma membrane and confirm that the interaction is dependent upon the ephrinB2 PDZ-binding motif and 35 amino acids within the TBC domain of TBC1d24. The question remained whether the ephrinB2/TBC1d24 association is direct or mediated through another partner protein.

### Dsh mediates the interaction between ephrinB2 and TBC1d24

**Fig. 2:**
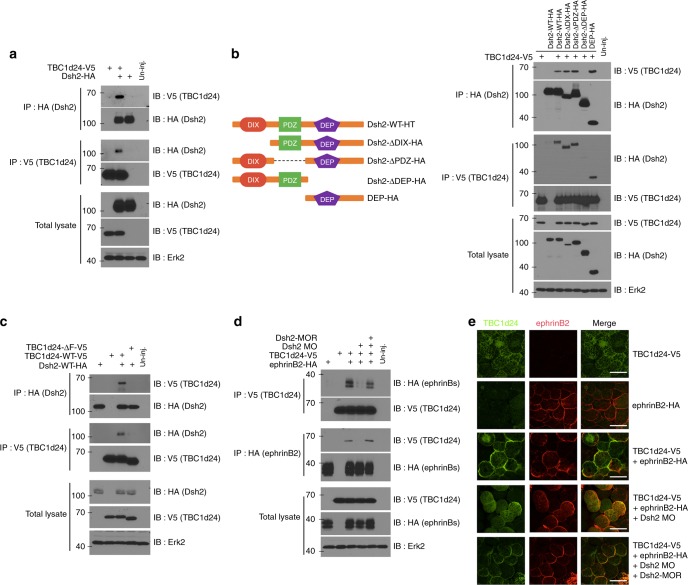
Dsh mediates the ephrinB2–TBC1d24 interaction. **a** Co-IP using gastrula embryos injected with RNA encoding TBC1d24-V5 and Dsh-HA (500 pg) shows that TBC1d24 binds to Dsh. **b** Illustration of the deletion mutants of Dsh. Co-IP using gastrula embryos injected with RNA encoding TBC1d24-V5 and domain deletion mutants of Dsh-HA shows that the DEP domain of Dsh associates with TBC1d24. **c** Co-IP using gastrula embryos injected with RNA encoding Dsh-HA and wild-type TBC1d24-V5 or the mutant TBC1d24-ΔF shows that the F region is required for an interaction with Dsh. **d** IP using gastrula embryos injected with RNA for TBC1d24-V5 and Dsh-HA along with Dsh2 morpholino oligonucleotides (Dsh2-MO) shows that knockdown of Dsh2 suppresses the interaction between ephrinB2 and TBC1d24, and this is rescued by the expression of MO-resistant Dsh2 (Dsh2-MOR). **e** IF of ectoderm from the embryos injected with the indicated MOs and RNAs shows that ephrinB2 (red) induces membrane localisation of TBC1d24 (green). This localisation is dramatically reduced by knockdown of Dsh2 and also rescued by introduction of Dsh2-MOR RNA. Bar, 50 μm

Dishevelled (Dsh) is a key molecule in Wnt signalling and is also known to be a major player in the Wnt/PCP pathway required for proper neural crest cell migration^18,39^. Previous studies demonstrated that Dsh interacts with the C-terminal PDZ-binding motif of ephrinB ligands^40–43^, which is also the motif required for the interaction with TBC1d24 (Fig. 1d). One possibility is that the ephrinB2–TBC1d24 interaction is mediated through an association with Dsh2. Co-IP analysis was performed with lysates from the embryos exogenously co-expressing tagged versions of TBC1d24 and Dsh2. The results clearly indicate the presence of Dsh2 in TBC1d24 immune-complexes and that of TBC1d24 in Dsh2 immune-complexes (Fig. 2a). In an IP analysis of Dsh2 from lysates of LS174T human colon carcinoma cells, TBC1d24 was detected in the Dsh2 immune-complexes (Supplementary Fig. 1c), indicating that the endogenous proteins are found associated. Co-IP analysis of Dsh2 domain-specific deletion constructs that were co-expressed along with TBC1d24 in the embryos indicates that the formation of a complex between Dsh2 and TBC1d24 was disrupted by deletion of the DEP domain in Dsh2 (Fig. 2b). A construct representing only the DEP domain associated with TBC1d24 (Fig. 2b). Together, the evidence indicates that the DEP domain is required for an interaction between Dsh2 and TBC1d24. Complementary Co-IP experiments were performed using TBC1d24 domain-specific deletion mutants (Supplementary 1d), as well as specific deletion mutants within the TBC domain (Fig. 2c). The results show that the F region within the TBC domain of TBC1d24 is required for the interaction with Dsh2 (Fig. 2c).

These IP results were consistent with the possibility that Dsh2 mediates the ephrinB2–TBC1d24 interaction, and in support of this hypothesis is the report showing that the DEP domain of Dsh2 is critical for an association with ephrinB1^42^. To test this concept, we employed Dsh2 anti-sense morpholino oligonucleotides (Dsh2-MO) to knockdown endogenous Dsh2 in the embryos. Dsh2 morphant embryos displayed a remarkable reduction in the ephrinB2–TBC1d24 interaction, which was recovered by exogenously re-expressing Dsh2 through injecting a MO-resistant Dsh2 RNA (Fig. 2d). As a further test of the requirement for Dsh2, we also examined the localisation of TBC1d24 in embryonic ectoderm in the Dsh2-MO injected embryos (Fig. 2e). Consistent with the Co-IP results, the Dsh2-MO reduced TBC1d24 membrane localisation, whereas Dsh2 RNA injection rescued the TBC1d24 localisation to the membrane in ephrinB2 co-expressing embryos (Fig. 2e). Taken together, our results show that Dsh2 mediates the ephrinB2–TBC1d24 association.

### Phosphorylation of ephrinB2 regulates the interaction with TBC1d24

**Fig. 3:**
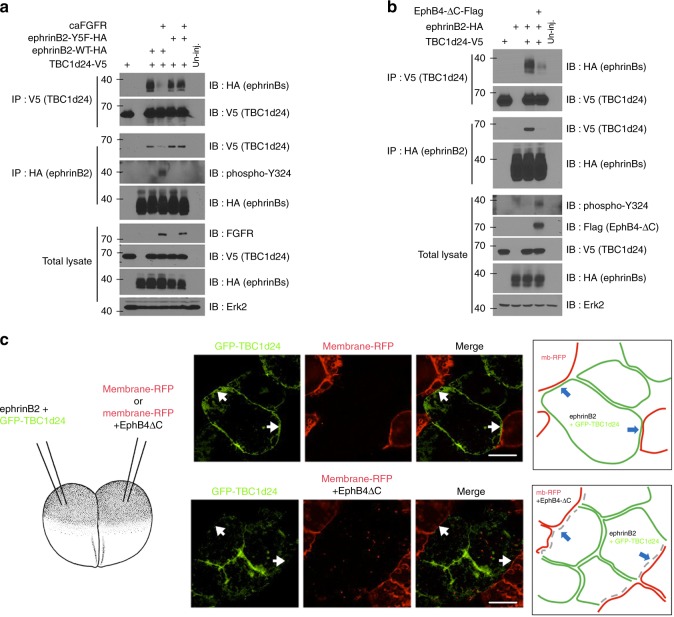
The interaction between ephrinB2 and TBC1d24 is disrupted by tyrosine phosphorylation. **a** Co-IP using gastrula embryos injected with RNA for TBC1d24-V5 and wild-type ephrinB2-HA or the ephrinB2 tyrosine mutant (Y5F) along with a constitutively active mutant of FGFR1 (caFGFR; 250 pg) shows that ephrinB2 phosphorylation suppresses the interaction between TBC1d24 and ephrinB2. **b** Co-IP using gastrula embryos injected with RNA encoding TBC1d24 and ephrinB2 wild type along with EphB4-ΔC (having a truncated cytoplasmic domain) (1 ng) shows that the interaction between ephrinB2 and the ectodomain of its cognate receptor EphB4 inhibits ephrinB2–TBC1d24 binding. **c** IF of ectoderm tissue excised from the embryos injected with the indicated RNAs shows that membrane localisation of GFP-TBC1d24 (green) in ephrinB2-expressing cells is dramatically reduced at the site of cell–cell contact with cells expressing both EphB4-ΔC and membrane-RFP (red). However, membrane localisation of GFP-TBC1d24 is unaffected by contact with only membrane-RFP-expressing cells. The white arrows indicate cell–cell contact regions. The cartoon depicts areas in the merged image. Bar, 20 μm

EphrinB1 can be tyrosine phosphorylated in response to binding the extracellular domain of a cognate Eph receptor^44,45^, the tight-junction-associated protein Claudin^46^ or in response to FGFR activation^47^. These phosphorylation events can regulate signalling partner associations and reverse signalling through the intracellular domain of ephrinBs^36^. Of note, phosphorylation of ephrinB1 is known to cause the disengagement of its physical association with Dsh^40,42^. Thus we tested whether the ephrinB2/TBC1d24 association is also regulated by tyrosine phosphorylation, as would be expected if Dsh were the mediator of the interaction. When a constitutively active FGFR1 (caFGFR) or an EphB4 receptor lacking the tyrosine kinase domain (EphB4-ΔC) was expressed in *Xenopus* embryos, ephrinB2 tyrosine phosphorylation was induced (Fig. 3a, b). Interestingly, immunoprecipitation and western blot analysis of ephrinB2 indicates that phosphorylation causes a marked reduction in its interaction with TBC1d24 (Fig. 3a, b), whereas a mutant ephrinB2 with all five conserved tyrosines changed to phenylalanine showed no alteration in its interaction with TBC1d24 (Fig. 3a). Together, these data indicate that phosphorylation induced by either binding of EphB or activation of the FGFR can disrupt the association between ephrinB2 and TBC1d24.

To test whether contact between ephrinB2 and its cognate receptor also results in inhibiting ephrinB2-induced membrane localisation of TBC1d24, ephrinB2 and green fluorescent protein (GFP)-TBC1d24 RNA were injected into one blastomere of a two-cell-stage embryo. The other blastomere was injected with membrane-red fluorescent protein (membrane-RFP) RNA singly or along with EphB4-ΔC RNA. The animal ectoderm region was dissected and observed under confocal microscopy at stage 10 (gastrula). TBC1d24 protein localised to the membrane at the point of cell–cell contact between the cells expressing both ephrinB2 and GFP-TBC1d24 and the cells expressing the membrane-RFP alone, as expected (Fig. 3c). In contrast, membrane localisation of TBC1d24 along the cell–cell contact region was significantly decreased when cells expressing both ephrinB2 and GFP-TBC1d24 were in contact with cells expressing EphB4-ΔC along with membrane-RFP (Fig. 3c). Although tyrosine phosphorylation disrupts the interaction between ephrinB2 and the Dsh/TBC1d24 complex, it was still possible that, upon contact with EphB4-ΔC-expressing cells, ephrinB2 is endocytosed and leads to loss of TBC1d24 membrane localisation. However, expression of an ephrinB2 tyrosine to phenylalanine substitution mutant (ephrinB2-Y2F) that precludes phosphorylation of the two tyrosines necessary for disengagement of Dsh^40^ maintained the membrane localisation of GFP-TBC1d24 at the site of cell–cell contact with EphB4-ΔC (Supplementary Fig. 1e). Taken together, our results demonstrate that TBC1d24 interacts with ephrinB2 via Dsh and is localised to the membrane. However, upon tyrosine phosphorylation, the association between ephrinB2 and TBC1d24 is disrupted leading to re-localisation of TBC1d24 from the plasma membrane to the cytoplasm.

### Loss of TBC1d24 function causes CNC cell migration defects

**Fig. 4:**
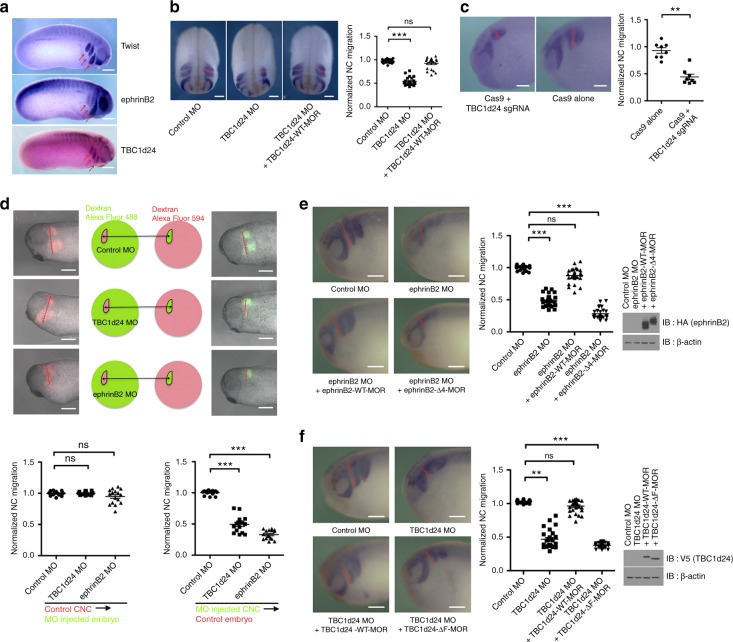
EphrinB2–TBC1d24 interaction regulates CNC cell migration. **a** Spatial expression pattern of *Twist*, ephrinB2 and TBC1d24. WISH of stage 24 embryos with probes for *twist* (a CNC marker), *ephrinB2* and *TBC1d24* (red arrows indicate CNC). Lateral view, anterior is right. **b** WISH using the *Twist* antisense probe in embryos injected with Control MO or TBC1d24MO alone or along with TBC1d24 MO-resistant RNA. MOs and/or RNAs were injected into the D.1.2 blastomere. The dotted red line denotes distance of CNC migration on the injected side of the tailbud stage embryo. Anterior/dorsal view. Quantification with non-parametric ANOVA (Kruskal–Wallis test), *P* *<* 0.0001. **c** WISH using the *Twist* antisense probe in F0 embryos injected with sgRNA for TBC1d24 and/or Cas9 protein. Dotted red line denotes distance of CNC migration. Quantification with Mann–Whitney test: **P = 0.0013, two-tailed). **d** Green fluorescein dextran (GFD)-labelled neural crest explant tissue from control, TBC1d24 or ephrinB2-depleted embryos was exchanged with normal neural crest explant tissue from red fluorescein dextran (RFD)-labelled embryos at stage 20 as indicated in the schematic (left). At the tailbud stage (right), it is observed that depletion of ephrinB2 or TBC1d24 causes neural crest cell migration defects in a tissue-autonomous manner. Quantification with one-way ANOVA, *P* < 0.0001. **e** WISH using the *Twist* antisense probe in the embryos injected with ephrinB2 MO alone or with MO-resistant wild-type or Δ4 ephrinB2 mutant RNA as indicated. Dotted red line denotes distance of CNC migration. Quantification with non-parametric ANOVA (Kruskal–Wallis test), *P* < 0.0001. Western blot shows the similar indicated protein expression. **f** WISH using twist probe in embryos injected with the indicated MOs and RNAs. The red arrow indicates the CNC migration differences between embryos. Western analysis shows that mutant and wild-type MO-resistant RNAs yield similar levels of protein expression. Quantification with non-parametric ANOVA (Kruskal–Wallis test), *P* < 0.0001; Bar, 200 μm. All histograms and scatterplots represent mean ± s.e.m from three biological repeats; Dunnett’s multiple comparison; **P < 0.01 and ***P < 0.001, ns: no statistical differences between groups

EphrinB2 is expressed in CNC cells of vertebrate embryos, including mouse, chick and frog^22,24,27,48^. We performed whole-mount in situ hybridisation (WISH) with probes for TBC1d24 and found that its expression overlaps with ephrinB2 in CNC cells and somites (Fig. 4a). To define the functional role of TBC1d24 in embryonic CNC, we designed a specific MO against TBC1d24 (TBC1d24 MO) and confirmed the ability of the MO to block translation of exogenous protein (Supplementary Fig. 2a). Interestingly, embryos injected at the two-cell stage with TBC1d24 MO show reduced pharyngeal pouch formation that is a typical phenotype induced by CNC cell migration defects at the tailbud stage (Supplementary Fig. 2b)^49^. To exclude possible indirect causes for the phenotype such as improper gastrulation or delayed neural tube closure, embryos were injected in the D.1.2 blastomere of 16-cell-stage embryos, which restricts the MO to the region giving rise to neural crest (Supplementary Fig. 2c). In these experiments, one side of the embryo has the progeny cells harbouring the MO, while the other side does not, acting as a control. WISH was performed using a probe for *Twist*, used as a marker for migrating neural crest. In the early neurula-stage embryos, neural crest induction was not altered by TBC1d24 MO and the muscle marker, MyoD, was also unchanged (Supplementary Fig. 2d). Of particular importance, in the tailbud-stage embryos, neural crest cell migration was suppressed in a MO dose-dependent manner (Fig. 4b and Supplementary Fig. 2e). This was also confirmed using a probe for Slug, another neural crest-specific marker (Supplementary Fig. 2f). The CNC migration defects were rescued by injection of MO-resistant wild-type TBC1d24 RNA, verifying that the defect was specific to reduced TBC1d24 (Fig. 4b and Supplementary Fig. 4f). To further validate the loss-of-function phenotype conferred by the TBC1d24 MO, we employed a knockout strategy using CRISPR/Cas9 (Supplementary Fig. 2g). TBC1d24 CRISPR-targeted embryos also showed similar CNC migration defects (Fig. 4c).

The ephrinB2 ligand is a transmembrane protein that is capable of bi-directional signalling, which prompted a test of whether TBC1d24- or ephrinB2-mediated effects were tissue autonomous or non-autonomous. At the 16-cell stage, TBC1d24 MO or ephrinB2 MO were injected along with a fluorescent tracer into the D.1.2 blastomere and incubated until stage 20 (when neural crest is migratory). Neural crest tissue was excised and transferred to a recipient embryo where the neural crest tissue had been previously removed (Fig. 4d and Supplementary Fig. 2h). Neural crest cells lacking either TBC1d24 or ephrinB2 failed to migrate in the wild-type-recipient embryos, whereas wild-type neural crest cells migrate normally even in recipient embryos in which TBC1d24 or ephrinB2 were depleted in the surrounding tissue (Fig. 4d). Moreover, morphant CNC explants injected with MO-resistant wild-type TBC1d24 or ephrinB2 RNA, respectively, had ventral migration of CNC restored (Supplementary Fig. 2h). These results strongly imply that ephrinB2 and TBC1d24 regulate neural crest cell migration in a tissue autonomous manner.

### An ephrinB2/TBC1d24 interaction is required for CNC migration

Since our biochemical and cell biological data established that ephrinB2 interacts with TBC1d24 and controls the localisation of TBC1d24, we tested whether the TBC1d24 and ephrinB2 interaction plays a significant role in CNC migration. Both ephrinB2 and TBC1d24 morphant embryos displayed abnormal CNC cell migration, which was rescued by expressing their wild-type counterparts (Fig. 4e, f, respectively). However, the expression of ephrinB2-Δ4, which cannot associate with TBC1d24, or the expression of TBC1d24-ΔF, which cannot interact with ephrinB2, failed to rescue their affiliated morphant defects (Fig. 4e, f, respectively). Western blot analysis shows that the expression of the interaction mutants was similar to their wild-type counterparts (Fig. 4e, f). These observations suggest that ephrinB2 and TBC1d24 interaction plays a critical role in proper neural crest cell migration.

### Rab35 interacts with TBC1d24 and regulates CNC migration

Skywalker is the Drosophila homologue of TBC1d24 that functions as a RabGAP for Rab35^50^. TBC1d24 is also known to regulate neuronal migration and maturation by negatively regulating activation of Arf6, which is involved in membrane trafficking^51^. In addition, a number of other Rabs are potential candidates for affecting or regulating CNC migration: Rab11 is associated with E-cadherin recycling in mammals and Fly^52^, while Rab5 and Rab7 have been implicated in lysosomal targeting of E-cadherin^53^. A limited Co-IP screen was performed with Arf6 and several Rabs in *Xenopus* embryos, and *Xenopus* TBC1d24 interacted with Rab5 and Rab35, but Arf6 showed only a weak interaction (Supplementary Fig. 3a). Co-IP analysis of lysates from the embryos expressing TBC1d24 mutants harbouring small deletions within the conserved TBC domain (delineated in Fig. 1c)^50,51^ indicated that the TBC1d24-ΔD mutant showed a significant reduction in the ability to bind Rab35, while the TBC1d24-ΔF mutant (which is unable to interact with ephrinB2) interaction was unchanged (Supplementary Fig. 3b). Unlike wild-type TBC1d24, expression of the TBC1d24-ΔD mutant failed to rescue CNC migration in TBC1d24 morphant embryos (Supplementary Fig. 3c). These findings strongly suggest that the binding of Rab35 to TBC1d24 plays a key role in proper CNC migration.

### TBC1d24 modulates CIL via regulating E-cadherin

**Fig. 5:**
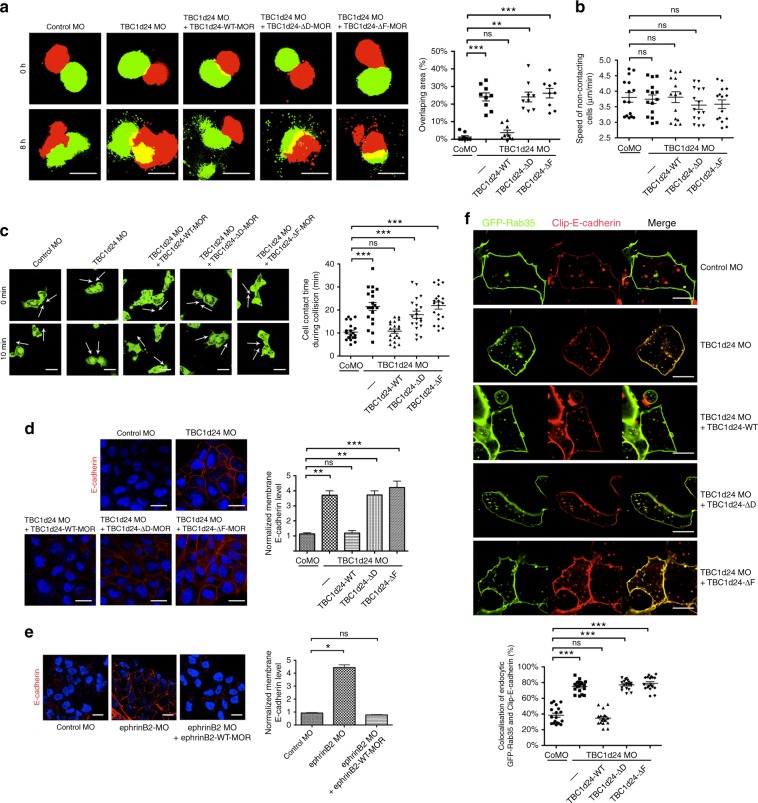
TBC1d24 modulates CIL via regulating E-cadherin. **a** Homotypic invasion assay. CNC explants injected with control MO or TBC1d24 MO alone or with the indicated TBC1d24 MO-resistant WT or mutant RNA and juxtaposed with RFD-injected CNC explants. Invasion is determined by the amount of explant overlap (yellow). Quantification with non-parametric ANOVA (Kruskal–Wallis test), *P* < 0.0001; Bar, 200 μm. **b** Histogram depicts ex vivo analysis of the speed of non-contacting CNC cells (μm/min) injected with the indicated MOs and RNAs. Quantification with one-way ANOVA, *P* = 0.8846. **c** Ex vivo migrating neural crest cell contact assay. The white arrows in the IF still images (from Supplementary Movie 1) of cells from CNC explants injected with the specified MOs and RNAs indicate the direction of migration at the indicated time of collision. The histogram indicates contact time during collisions of cells injected with control or TBC1d24 MOs alone or with the indicated TBC1d24 RNAs. Data represents means ± s.e.m from four biological repeats. Quantification with one-way ANOVA, *P* < 0.0001; Bar, 20 μm. **d** IF of CNC tissue using anti-E-cadherin Ab (red) from the embryos injected with control or TBC1d24 MOs alone or with the indicated TBC1d24 RNAs. Histogram represents relative E-cadherin levels. Quantification with non-parametric ANOVA (Kruskal–Wallis test), *P* < 0.0001; Bar, 20 μm. **e** IF of CNC tissue with an E-cadherin Ab (red) from the embryos injected with control or ephrinB2 MOs alone or with MO-resistant ephrinB2 RNA. Histogram represents relative E-cadherin levels. Quantification with non-parametric ANOVA (Kruskal–Wallis test), *P* < 0.0001; Bar, 20 μm. **f** IF of CNC explants expressing GFP-Rab35 (green) and Clip-tagged E-cadherin (red) also injected with control or TBC1d24 MOs alone or with the indicated TBC1d24 RNAs. Co-localisation displayed as yellow area. Histogram represents the percentage of overlapping signal in endocytic vesicles. Quantification with one-way ANOVA, *P* < 0.0001. Bar, 10 μm. All histograms and scatterplots represent mean ± s.e.m from three biological repeats unless otherwise indicated; Dunn’s multiple Comparison, *P < 0.05, **P < 0.01 and ***P < 0.001, ns: no statistical differences between the groups

One major contributor to the appropriate migration of CNCs is a process known as CIL, in which cells halt migration in response to contact with another cell. In CNC cells, homotypic collisions or contact between CNC cells leads to retraction of cell protrusions, repolarisation and cells move away from each other. To examine how loss of TBC1d24 function causes neural crest cell migration defects, we dissected migratory neural crest tissue from stage 20 embryos that were previously injected with MOs and RNAs, as indicated in Fig. 5a. The neural crest tissue explants injected with either green or red fluorescent dextran (GFD or RFD, respectively; as a lineage tracer) were placed on fibronectin-coated plates and subjected to an invasion assay that tests whether the explants have lost CIL and thus leads to one explant mixing or invading into the other explant. Unlike control MO-injected neural crest tissue (green), TBC1d24-depleted neural crest tissue (green) invaded the uninjected neural crest tissue (red), demonstrating that homotypic CIL is compromised (Fig. 5a). Co-injecting wild-type TBC1d24 RNA into the TBC1d24-depleted explant blocked invasion and restored CIL (Fig. 5a). EphrinB2-depleted neural crest tissue (green) also invaded the uninjected neural crest tissue (red), which was rescued by expressing the wild-type form (Supplementary Fig. 3d). However, the expression of ephrinB2-Δ4, which cannot bind to TBC1d24, failed to block the invasive behaviour (Supplementary Fig. 3d). Neither TBC1d24-ΔD nor TBC1d24-ΔF expression in TBC1d24-depleted neural crest explants (green) rescued CIL, as evidenced by the invasive behaviour (Fig. 5a). These data strongly indicate that TBC1d24 requires an interaction with both Rab35 and ephrinB2 to preserve homotypic CIL.

An additional test of whether these interactions are critical to promote homotypic CIL was initiated. An ex vivo CNC cell migration assay was performed on similarly injected embryos whose neural crest was excised and dissociated to examine single-cell migration. In this assay, homotypic collisions between CNC cells that display CIL should lead to retraction of cell protrusions, repolarisation and cell migration away from each other. CNC cells from the control MO-injected embryos and TBC1d24-depleted cells that had not collided with other CNC cells showed no significant differences in migration direction or speed (Fig. 5b). This was also true of those CNC cells expressing wild-type TBC1d24, TBC1d24-ΔD or TBC1d24-ΔF interaction mutants (Fig. 5b). In contrast, there was a significant increase in contact time during cell collisions between CNC cells lacking TBC1d24 (Fig. 5c; Supplementary movie 1), which is a hallmark of loss of CIL^54^. Consistent with the invasion assays, re-expression of wild-type TBC1d24 in TBC1d24-depleted cells led to a restoration of normal contact times between CNC cells (Fig. 5c; Supplementary movie 1). Of note, in the TBC1d24-depleted cells, expression of the TBC1d24 mutants that are unable to interact with Rab35 or ephrinB2 failed to restore normal contact times (Fig. 5c; Supplementary movie 1). Taken together, these results suggest that TBC1d24 regulates homotypic CIL during neural crest cell migration.

It has been reported that CNC cells expressing E-cadherin remain in contact, while cells expressing N-cadherin undergo cell repolarisation and repulsion^3^. It was shown that overexpression of E-cadherin inhibits CIL behaviour after cell–cell collision^3^, and this is very similar to our results with the TBC1d24-depleted CNC cells (Fig. 5c; Supplementary movie 1). Furthermore, Rab35, which is a substrate of TBC1d24, is known to regulate E-cadherin recycling in the mammalian cell lines^31,55^. To assess effects on E-cadherin levels, neural crest tissue from the embryos injected with RNA encoding mutants of the three potential Arf/Rabs (Arf6, Rab5 and Rab35) that interact with TBC1d24 (Supplementary Fig. 3a) were dissected at stage 20 and immunostained for E-cadherin (Supplementary Fig. 3e). GFP control-expressing CNC cells displayed a low level of E-cadherin, as did those expressing a constitutively active or inactive Arf6. Overexpression of a constitutively active form of Rab35 (Rab35 Q67N) increased E-cadherin levels, while the inactive form of Rab35 (Rab35 S22N) reduced E-cadherin levels in *Xenopus* CNC cells (Supplementary Fig. 3e). Not surprisingly, the opposite result was observed with Rab5 mutants (Supplementary Fig. 3e). Rab5 is generally involved in early endocytosis from the membrane^56^, while Rab35 is mainly concerned with recycling to the membrane. Thus E-cadherin levels were only elevated in the active Rab35-expressing or inactive Rab5-expressing CNC cells.

Since elevated levels of E-cadherin are associated with inhibition of CIL in CNC cells^3^, we tested whether depletion of ephrinB2 or TBC1d24 also yields an increase in E-cadherin levels. Both TBC1d24 MO and ephrinB2 MO bearing neural crest tissue displayed elevated E-cadherin levels at the cell membrane, and this increase was blocked by re-expression of the wild-type counterparts of TBC1d24 or ephrinB2 (Fig. 5d, e). In the TBC1d24-depleted CNC cells, expression of the inactive Rab35 blocked the enhanced E-cadherin expression, while inactive Rab5 did not, supporting the data indicating that TBC1d24 exerts its regulation of E-cadherin through Rab35 (Supplementary Fig. 3f). N-cadherin levels did not show any significant changes in TBC1d24- or ephrinB2-depleted neural crest tissue(Supplementary Fig. 3g). Expression of the Rab35 or ephrinB2 interaction mutants of TBC1d24 failed to decrease the enhanced E-cadherin levels observed with the loss of TBC1d24 (Fig. 5d), strongly suggesting that the interaction between TBC1d24 and these proteins is critical for regulating surface E-cadherin levels.

A more direct assessment of whether TBC1d24 is responsible for maintaining a low level of recycling of E-cadherin to the CNC membranes was performed. We expressed Clip-tagged E-cadherin that was later incubated in a cell non-permeable substrate to label surface E-cadherin. Neural crest tissue bearing the TBC1d24 MO showed a noticeably higher abundance of vesicles and plasma membrane regions that were positive for both Rab35 and E-cadherin (observed as yellow in the merged image in Fig. 5f; Supplementary movie 2), when compared to the control MO-injected CNC (observed as more distinct red and green areas in the merged image in Fig. 5f; Supplementary movie 2). This data is indicative of greater co-localisation of Rab35 and E-cadherin in the absence of TBC1d24, as would be expected if more E-cadherin is cycling to the membrane. When wild-type TBC1d24 was re-expressed in the TBC1d24 MO bearing CNC cells, co-localisation between Rab35 and E-cadherin was reduced, similar to control MO-injected CNC (Fig. 5f; Supplementary movie 2). Unlike wild-type TBC1d24, re-expressing either of the interaction mutants (TBC1d24-ΔD or TBC1d24-ΔF) failed to restore the separate localisation of Rab35 and E-cadherin (Fig. 5f; Supplementary movie 2). In addition, ephrinB2 knockdown significantly increased the levels of co-localisation between Rab35 and E-cadherin when compared the control MO-injected CNC (Supplementary Fig. 3h; Supplementary movie 3), which was inhibited upon re-expression of wild-type ephrinB2. However, expression of ephrinB2-Δ4, which cannot associate with TBC1d24, failed to decrease the co-localisation (Supplementary Fig. 3h; Supplementary movie 3). Taken together, our results indicate that the interaction of TBC1d24 with ephrinB2 and Rab35 is critical for regulating E-cadherin recycling.

### EphrinB2 and EphB4 regulate proper CNC cell migration

**Fig. 6:**
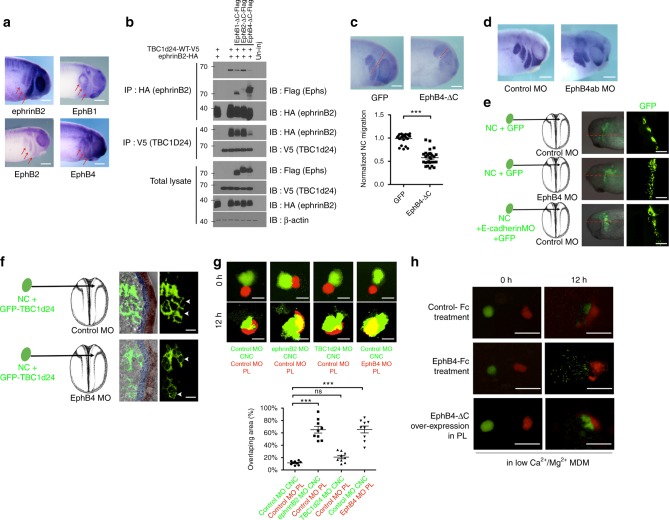
EphrinB2 and its cognate Eph receptor, EphB4, regulate appropriate CNC cell migration. **a** WISH shows ephrinB2 and EphB2 expression in neural crest cells and EphB1 and EphB4 in boundary of CNC streams (red arrows). Bar, 200 μm. **b** Co-IP and western blot using gastrula embryos injected with TBC1d24 RNA and ephrinB2 wild-type RNA along with truncated receptor EphB1-ΔC, EphB2-ΔC or EphB4-ΔC RNAs (1 ng each). **c**, **d** WISH using *Twist* antisense probe in the embryos injected into the D.1.2 blastomere with the indicated MOs or RNAs (red dotted lines indicate extent of CNC migration). Histogram depicts relative CNC migration (quantification with Mann–Whitney test: ***P = 0.0004, two-tailed). Bar, 200 μm. **e** Neural crest tissues injected with GFP RNA alone or along with E-cadherin MO were dissected and transferred into EphB4 MO or control MO-injected embryos as indicated. Confocal microscopic images of embryos sectioned along the red lines and the neural crest streams displayed. Bar, 200 μm. **f** GFP-TBC1d24 expressing neural crest tissues were dissected and transferred into EphB4 MO- or control MO-injected embryos. Embryos were sectioned and a magnified image shows that membrane localisation of GFP-TBC1d24 at the NC (neural crest)–PL (placode) contact site (the white arrow heads). Blue, placodes; brown, superficial ectoderm. Bar, 20 μm. **g** Heterotypic NC-PL invasion assay. Embryos were injected with the indicated MOs, and explants excised and placed juxtaposed. IF of GFD-labelled neural crest explants injected with the indicated MO (in green) juxtaposed to RFD-labelled placode explants injected with the indicated MO (red). Invasion of CNC into placode denoted by yellow area in image and depicted in histogram. Quantification with one-way ANOVA, *P* < 0.0001; bar, 400 μm. **h** CNC chemoattraction assay. CNC (injected with GFD—green) or placode [(PL) injected with RFD—red] tissues were dissected at stage 20, placed 400 μm apart and incubated in low Ca^2+^/Mg^2+^ medium for 12 h and treated with the indicated clustered Fc or injected with EphB4-ΔC RNA and imaged. Bar, 400 μm. All histograms and scatterplots represent mean ± s.e.m from three biological repeats; Dunn’s multiple Comparison, ***P < 0.001, ns: no statistical differences between the groups

Having found that overexpression of EphB4 disrupts the ephrinB2/TBC1d24 interaction (Fig. 3b, c), we examined how the Eph/ephrin interaction may regulate proper CNC cell migration. A WISH survey of potential cognate Eph receptors showed that EphB4 and EphB1 transcripts were found in the tissue that borders the neural crest stream, whereas EphB2 is expressed within the neural crest cells, much like ephrinB2 (Fig. 6a). Previous studies demonstrate that ephrinB2 displays more robust interactions with EphB4 than EphB2^57^, and our Co-IP results confirm this trend (Fig. 6b). To assess whether binding to the extracellular domain of specific cognate EphB receptors affects the ephrinB2/TBC1d24 interaction, we introduced truncated versions of EphB1 or EphB2 or EphB4 (EphB1-ΔC, EphB2-ΔC, EphB4-ΔC) into the embryos. These constructs lack the cytoplasmic domain to ensure that phosphorylation of ephrinB2 is induced by receptor binding and not by their kinase activity. The EphB4 receptor displayed the most prominent interaction with ephrinB2, followed by EphB1, and EphB2 being the weakest (Fig. 6b). Consistent with these binding preferences, EphB4 markedly disrupted the ephrinB2/TBC1d24 interaction in Co-IPs (Fig. 6b), and EphB2 had little effect (Fig. 6b). The biochemical, ex vivo and in vivo data strongly support a model where ephrinB2 interacts with TBC1d24 via Dsh, and this leads to proper localisation of Rab35 to regulate surface E-cadherin levels within the CNC cells. Moreover, upon contact with the cognate EphB4 receptor, tyrosine phosphorylation of ephrinB2 leads to disengagement of the complex (Supplementary Fig. 4a).

An ex vivo test of this concept was performed by treating neural crest explants that were plated on fibronectin with either clustered EphB4-Fc or Control-Fc. EphB4-Fc treatment significantly suppressed dispersion of CNC cells from the neural crest explant during a 12 h period, when compared to Control-Fc-treated tissue (Supplementary Fig. 4b). As expected from the model (Supplementary Fig. 4a), EphB4-Fc treatment of CNC explants increased E-cadherin levels in CNC cells (Supplementary Fig. 4c). These data indicate that contact of ephrinB2-expressing neural crest cells with the EphB4 ectodomain inhibits CNC migration via increasing E-cadherin levels.

Proper neural crest cell migration is critically dependent upon the control of E-cadherin levels within the CNC^21,58,59^. To assess whether the regulatory mechanism established in ex vivo assays may also occur in vivo, we ectopically expressed EphB4-ΔC in neural crest cells and examined the pattern of CNC migration through WISH by probing for *Twist* (Fig. 6c). The mis-expression of the EphB4-ΔC protein considerably inhibited neural crest cell migration, when compared to the GFP RNA-injected controls (Fig. 6c). One prediction from the model (Supplementary Fig. 4a) is that loss of EphB4 expression in the tissue surrounding the CNC streams may lead to mixing of the streams as a result of increased E-cadherin levels. Thus we injected EphB4 MO that effectively blocks exogenous EphB4 expression (Supplementary Fig. 4d) into the D.1.2 blastomere to deplete EphB4 in the CNC and surrounding tissues (Fig. 6d). Consistent with the prediction, the CNC migration streams mixed in the EphB4-depleted embryos and not in the control MO-injected embryos (Fig. 6d). As a further test of the model, we transplanted GFP-expressing CNC cells into host embryos that harboured either control MO or EphB4 MO and examined cross-sections through the CNC streams (Fig. 6e). CNC cells transplanted into EphB4 MO-injected host embryos displayed a complete lack of collective cell migration, similar to CNC cells that were depleted of E-cadherin and transplanted into control MO-injected hosts (Fig. 6e). Another expectation from the model is that membrane localisation of TBC1d24 would be disrupted in the normal developmental context when CNC tissue contacts the surrounding placode tissue where EphB4 is endogenously expressed. Therefore, we transplanted GFP-TBC1d24-expressing CNC into either wild-type or EphB4 MO-injected embryos (Fig. 6f). At the site of contact between CNC cells and surrounding placodal cells, the membrane localisation of TBC1d24 was markedly reduced (Fig. 6f). However, in a host embryo depleted of EphB4, clear membrane localisation of TBC1d24 was found at the site of contact between the CNC and placode (Fig. 6f; white arrow heads). Collectively, these data indicate that in CNC, ephrinB2 controls E-cadherin levels through the interaction with Dsh and TBC1d24. Furthermore, this interaction can be regulated in a tyrosine phosphorylation-dependent manner upon binding the cognate Eph receptor.

An ex vivo invasion assay was performed as an additional test of the influence of EphB4 on the interaction between CNC and placode tissue. GFP-expressing wild-type CNC explants were placed adjacent to control wild-type RFP-expressing placode explants on a fibronectin-coated substrate. The CNC cells repulsed the placode tissue without mixing with or invading the tissue (Supplementary movie 3). However, when the placode tissue was depleted of EphB4, the CNC tissue contacted and subsequently invaded the EphB4-depleted placode tissue (Supplementary movie 4). These data clearly indicate that EphB4 in the placode is critical to maintain separation of the CNC and placode tissue, which is a consistent with the conventional EphB4/ephrinB2 repulsive activity^60^.

Although we have established that the ephrinB2/TBC1d24 interaction is critical for homotypic CIL within the CNC, it was less clear whether it has a role in heterotypic CIL (CNC/placode). Therefore, we performed neural crest tissue explant invasion assays on a fibronectin substrate using either GFD (neural crest) or RFD (placode) as lineage tracers. Unlike control CNC, ephrinB2-depleted CNC tissue invaded the placode explant (Fig. 6g). Of particular interest, TBC1d24-depleted neural crest tissue did not display significant invasive activity into the placode tissue (Fig. 6g), demonstrating that heterotypic CIL (CNC/placode) is not compromised to a substantive degree. In contrast, EphB4 MO-containing placode tissue allows CNC explant invasion into the placode tissue (Fig. 6g). Together, these data indicate that TBC1d24 is not a major contributor to heterotypic CIL between CNC and placode, strongly suggesting that the repulsion between ephrinB2 in the CNC and EphB4 in the placode are critical for this process.

Eph–ephrinB reverse signalling is also known to regulate G protein-coupled chemoattraction^22^. Thus we tested whether the EphB4/ephrinB2 interaction may regulate chemotaxis of CNC towards the placode. To this end, we excised neural crest explants that were injected with GFD and placode explants containing RFD. These CNC migratory-stage explants were placed near each other on fibronectin-coated plates in a low calcium media for 12 h. This low Ca^2+^/Mg^2+^ media allows an assessment of chemotaxis in the absence of effects on homotypic cell–cell adhesion that might be exerted by an increase in surface E-cadherin, resulting from EphB4-Fc treatment of these explants. Explants treated with control-Fc showed the expected directional movement of CNC explant cells towards the SDF-1-secreting placode explants^11^ (Fig. 6h, Supplementary Movie 5). Strikingly, EphB4-Fc treatment suppressed the directional migration of CNC cells towards the SDF-1-expressing placode and caused the CNC cells to move randomly (Fig. 6h, Supplementary movie 5). This is consistent with ephrinB2 within the CNC inhibiting the activity of the SDF-1 receptor (C-X-C chemokine motif receptor 4 (CXCR4)) upon engagement with the cognate EphB receptor, as has been shown for cerebellar granule cell movement^61^. Similar to control explants, CNC explants exhibited directional migration towards the EphB4-ΔC-expressing placode explants. This data indicate that the clustered soluble EphB4 treatment inhibits the ephrinB2-expressing CNC response to chemoattractant from the placode but does not exert an effect on the placode explant (Fig. 6h, Supplementary movie 5). Collectively, these data suggest that the ephrinB2/TBC1d24 interaction is critical to homotypic CIL but is not a major contributor to heterotypic CIL between the CNC and placode. Moreover, EphB4 expressed in the placode exerts its influence upon contact with ephrinB2-expressing CNC cells mainly through repulsive activity and the inhibition of the CNC response to chemoattractant.

## Discussion

CIL is a crucial process for many essential embryological events, including collective cell migration and boundary formation, while loss of CIL plays a critical role in pathological states, such as cancer metastasis^54^. CIL is the normal procedure by which a cell halts movement or alters its direction upon collision with a neighbouring cell. One important developmental process where CIL is involved is CNC migration.

Neural crest cells are multipotent progenitor cells that, subsequent to dispersing from the neuroepithelium, migrate initially in three distinct streams and further branch off into other regions, including the prospective face, branchial arches, heart and gut^11^. There are many factors leading CNC cells to the correct locations^11^. However, it is cell–cell interactions that represent a critical mainspring for CNC migration through establishing cell polarisation and activating the PCP signalling pathway. As CNC cells start to migrate, the cells at the front of the group experience CIL at the rear and polarise toward the open front of the collective. During this movement, there is a loosening of the space between the collective cells, allowing for polarisation towards the collective front, and can thus be responsive to the attractant signals from the surrounding tissue.

Alterations in cadherin levels including E-cadherin, N-cadherin, cadherin-11 and protocadherins can exert substantive effects on CNC migration^1,3,4,6,62,63^. E-cadherin is highly expressed in the pre-migratory CNC cells, but E-cadherin downregulation is required for appropriate CIL behaviour during CNC cell migration^3,11^. Here we found that knockdown of ephrinB2 or TBC1d24 caused elevation of E-cadherin levels in CNC cells at the migratory stage, whereas levels of N-cadherin remained unchanged, resulting in a reduced CIL response in CNC cells both in vitro and in vivo (Fig. 5c–e, Supplementary Fig. 3e and f). Moreover, our data support a mechanistic model for how ephrinB2 may regulate CIL in CNC cells via TBC1d24. TBC1d24 interacts with ephrinB2 through the known association of ephrinB2 and Dsh^40–43^, as indicated in Fig. 2. Moreover, the interaction between ephrinB2 and TBC1d24 localises TBC1d24 to the membrane, and depletion of endogenous Dsh in *Xenopus* embryos disrupts this localisation (Fig. 2e). The ephrinB2/Dsh/TBC1d24 complex modulates E-cadherin levels through regulating Rab35 activity. A previous report implicated Arf6 as a substrate of TBC1d24 that regulates neuronal cell migration^51^. However, our results showed that, in *Xenopus* embryos, overexpression of Arf6 or a constitutively active form of Arf6 did not affect E-cadherin levels (Supplementary Fig. 3e) and Arf6 showed only a weak interaction with TBC1d24 (Supplementary Fig. 3a). We demonstrated that, although both Rab5 and Rab35 can robustly interact with TBC1d24 in CNC cells (Supplementary Fig. 3a), only inactive Rab35 induces elevated E-cadherin levels associated with inhibition of CIL (Supplementary Fig. 3e).

Eph–ephrin signalling has been implicated in regulating neural crest cell migration^22,23,25–27^, and Eph–ephrin interactions transduce signals in CNCs that can affect cell–cell repulsion or cell–cell adhesion^60^. These interactions result in preventing the mixing of CNC streams through contacts between CNCs and the surrounding tissue^64^. Our results showed that, in *Xenopus* CNC, the combination of the EphB4 receptor in the surrounding tissue and the ephrinB2 ligand in the CNC cells plays a role in three aspects of directed collective migration: (1) repulsion between CNC cells and the placode that are independent of TBC1d24; (2) SDF-1-directed chemotaxis of CXCR4 containing CNC cells towards the SDF-1 ligand in the placode; and (3) loss of homotypic CIL in CNC cells upon contact with the EphB-containing placode. Interestingly, the relevance of Eph–ephrin signalling in CIL has been studied in metastatic cancer cells, where it was found that homotypic collisions between two prostate cancer cells displayed CIL that was facilitated by EphA/Rho/Rho kinase signalling. Upon heterotypic collisions between prostate cancer cells and fibroblasts, the CIL response was dramatically decreased owing to enriched levels of EphB3 and EphB4 in the prostate cancer cells^65^.

In CNC cells, homotypic CIL among neural crest cells is postulated to restrict the protrusion generation between neighbouring cells, thus polarising the whole group for a common directional movement^4^. There are several steps to CIL including an initial cell–cell contact and adhesion stage^54^, and this may be where ephrinB2/Dsh/TBC1d24 interactions have the most influence on CIL, through maintaining low E-cadherin levels at the cell surface in a manner independent of the EphB receptor. SDF-1 secreted from placode cells attracts neural crest cells, but placodal cells undergo CIL in response to contact with the neural crest cells, resulting in repulsion away from the neural crest cells^62^. Our study indicates that the homotypic CIL among ephrinB2-expressing CNC cells in conjunction with SDF-1-driven attraction towards the placode cells represents a major force promoting CNC collective migration to the placodes. Upon engagement with the EphB4-expressing placode cells, ephrinB2-expressing CNC cells suppress the chemotactic response and undergo an Eph/ephrin repulsion that is independent of TBC1d24.

In summary, our data provide evidence that, in CNC cells of *Xenopus* embryos, the ephrinB2 transmembrane ligand interacts with TBC1d24, a negative regulator of Rab 35. The scaffold protein Dsh mediates the association between ephrinB2 and TBC1d24 and leads to re-localisation of TBC1d24 to the membrane where it negatively regulates Rab 35-dependent endosome recycling of E-cadherin. This regulation maintains a low level of E-cadherin expression at the CNC cell membrane, which allows for proper CIL and migration of these cells. The interaction between the ephrinB2 ligand in the CNC and its cognate EphB4 receptor in the surrounding tissue induces tyrosine phosphorylation of the intracellular domain of ephrinB2, leading to disengagement of Dsh and TBC1d24, induction of repulsive activity, inhibition of the chemotactic response and ceasing the movement of these cells.

## Methods

### Plasmids and reagents

The cDNA clone that encodes full-length TBC1d24 was obtained from Source BioScience (GenBank ID: BC128694). The sequences of MOs are as follows; ephrinB2 MO, 5′-GAGTCCCCGCTCAGTGCCATGATCT-3′^66^; TBC1d24 MO, 5′- AACGGCCATATTCAGCTTCATCCAT-3′; EphB4 MO, 5′-GGAGGAGCAGCTATAAATTGATCCA-3′; Dsh2 MO, 5′-TCACTTTAGTCTCCGCCATTCTGCG-3′^42,67^; and E-cadherin, 5′-AACCAGGGCCTCTTCAACCCCATTG-3′^68^. Various HA-tagged mutants of ephrinB2 (∆4, ∆6, ∆10, ∆16, ∆19 and Y5F) and the Dsh2 deletion mutants have been reported^42,66^. V5-tagged deletion mutants of TBC1d24 (∆N, ∆C, ∆TLD, ∆TBC and ∆A–∆F) were generated using the QuikChange II Site-Directed Mutagenesis Kit. To test whether tyrosine phosphorylation affects the ephrinB2–TBC1d24 interaction, FGFR1 KE (FGFR1 K562E), FGFR1 KD (FGFR1 C289R/K420A) and EphB4 ∆C–Flag (amino acids 1–596) were generated in the pCS2+ vector^40^. Clip-tag was synthesised and amplified by PCR. The Cadherin prodomain (amino acids 30–150) was replaced by an amplified Clip-tag in the pCS2-E-cadherin clone.

### Embryonic injections

We obtained *Xenopus* embryos using standard methods^69^. Using the SP6 mMessage mMachine Kit (Ambion), we produced capped mRNAs that were microinjected into embryos at the indicated dose, as described in the figure legends. For the rescue of MO effects, MO-resistant mRNA was synthesised. In the case of TBC1d24-MOR, eight nucleotide substitutions were generated in wobble codons subsequent to the ATG start codon (Supplementary Fig. 2a). MOs and mRNAs were microinjected into the animal pole region in one-cell-stage embryos or the D.1.2 blastomere at the 16-cell stage as indicated in Supplementary Fig. 2c. All animal studies were approved by the NCI at Frederick Animal Care and Use Committee [ACUC].

### IP and western blot analysis

*Xenopus* embryo lysates or LS174T cell (ATCC) lysates were prepared with ice-cold TNSG buffer (20 mM Tris-HCl pH 7.5, 137 mM NaCl and 1% NP-40). IPs were performed for 8 h with 25 embryo equivalent extracts using monoclonal Anti-Flag, HA or V5-agarose (Sigma-Aldrich). Endogenous protein IPs were conducted for 8 h with LS174T cell extracts using an anti-ephrinB2 antibody (1:500, C-18, Santa Cruz Biotechnology) or anti-Dsh2 antibodies (1:500, 3216, Cell Signaling). Western blot analysis was performed using anti-Flag–horseradish peroxidase (HRP)-conjugated (1:5000, A8592, Sigma), anti-HA–HRP-conjugated (1:5000, 12013819001, Roche), anti-phosphotyrosine–HRP-conjugated (1:1000, 4G10, Upstate Biotechnology), anti-ephrinB2 (1:1000, SAB4300456, sigma), and anti-ERK2 (1:1000, Santa Cruz Biotechnology) antibodies.

### Immunofluorescence microscopy

Animal cap explants were collected at stage 10.5 and immunofluorescence microscopy was carried out using standard protocols^42,66^. The following primary antibodies were used: anti-V5 (1:500, G189, ABM), anti-HA (1:1000, C29F4, Cell Signaling Technology), anti-E-cadherin (1:10, 5D3, DSHB) and anti-N-cadherin (1:20, MNCD2, DSHB).

### In vitro neural crest cell migration assay

*Xenopus* CNC cells were labelled with GFD that was injected into the D.1.2 blastomere at the 16-cell stage. For single-cell collision assays, CNC cells were briefly dissociated in Ca^2+^/Mg^2+^-free Danilchick medium^3^ and then cultured on fibronectin-coated plates (100 µg/ml) in Modified Danilchick Medium (MDM, NaCl 53 mM, Na_2_CO_3_ 11.7 mM, K Gluconate 4.27 mM, MgSO_4_ 2.035 mM, CaCl_2_ 1.32 mM, bovine serum albumin 0.1%, adjust pH to 8.3 with 1 M Bicine)^70^. Time-lapse images were taken using confocal microscopy. Control-Fc and EphB4–Fc (R&D Systems) were clustered using human immunoglobulin^63^.

### Co-localisation of GFP-Rab35 and Clip-E-cadherin

MOs and RNAs were injected with RNAs of GFP-Rab35 (50 pg) and Clip-E-cadherin (25 pg) into the D.1.2 blastomere at the 16-cell stage. GFP-positive neural crest tissues were dissected from stage 20 embryos and incubated with Clip-tag substrate (Non-cell-permeable, Clip-Surface™ 547) for 1 h at room temperature in 1× MBS. After brief washing with MDM, the neural crest tissue explants were placed on fibronectin-coated plates in MDM and incubated for 30 min to allow the explants to attach to the plates. Time-lapse images were taken using confocal microscopy (Zeiss LSM710/780).

### Whole-mount in situ hybridisation

*Xenopus* embryos were collected at stage 16 and 26^69^ for hybridisation with the *Twist*, *slug* and *myoD* probes. Embryos were injected with Dextran alexa-488 and various mRNAs or MOs to distinguish the injected side of embryos. The embryos were then processed for WISH using standard methods^71^.

### TBC1d24 knockout using CRISPR/CAS9

For guide RNA design, the design tool CRISPRdirect (https://crispr.dbcls.jp) application was used to scan the genome sequence for suitable CAS9 target sequences including a PAM site. As shown in Supplementary Fig. 2g, a sequence spanning 379–401 in the first exon of the TBC1d24 (5′- TGCCGTACTGTGACCCCCGA-3′) on the reverse strand was selected since no off-target effects were predicted. Single-guide RNA (sgRNA) template construction, in vitro transcription of sgRNA, microinjection and genotyping were performed as described in Nakayama et al. (2014)^72^. Small sections of the tail (2 mm) were dissected from F0 embryos and then analysed to verify mutagenesis by the direct sequencing of PCR amplicons assay^73^. Anterior parts of F0 embryos were used for WISH to assess CNC cell migration.

### Delaunay triangulation

Individual cells were selected and used to build triangles between the neighbouring cells using the Delaunay triangulation application of Matlab software. Then each triangular shaped area was calculated. An average triangular area was normalised by the mean of the Control-Fc-treated batch of each experiment.

### Invasion assay and chemoattraction assay

MOs and RNAs were injected along with GFD into the D.1.2 blastomere at the 16-cell stage, and neural crest tissue explants from GFD embryos were excised at stage 20. Neural crest tissue explants or placode tissue explants from the embryos injected with RFD were excised and placed next to the GFD-containing explants on fibronectin-coated plates in MDM and observed. After a 12 h incubation, the explants of the experimental group invaded (due to loss of CIL) into the normal RFD explants as demonstrated by the increased area of overlap between the explants (yellow area, white arrows). For the chemoattraction assay, GFD-injected neural crest explants and RFD-injected placode explants were dissected at stage 20 and placed about 400 μm apart and incubated in low Ca^2+^/Mg^2+^ MDM^62^. Control-Fc and EphB4–Fc (R&D Systems) were clustered using human immunoglobulin^74^.

### Statistical analysis

Sample size was determined as indicated in the figures and specific statistical method was not used. Dead cells and embryos were excluded from all experimental analysis. Embryos which have mis-targeted injection also were excluded (the target injection was confirmed by co-injection of lineage tracer (GFP RNAs, GFD or RFD). All experiments were performed blinded with order of testing randomised. ImageJ program was used for all quantification. All experiments were performed for at least three independent times. Normality of data was tested using Kolmogorov–Smirnov’s test, D’Agostino and Pearson omnibus normality test and Shapiro–Wilk normality test using Prism5. The data were considered normal if found as normal by all three tests. Data sets following a normal distribution were compared with Student’s *t* test (two-tailed, unequal variances) or a one-way analysis of variance (ANOVA) with Dunnett’s multiple comparisons post-test in Prism5. The data that did not follow a normal distribution were compared using Mann–Whitney’s test or a non-parametric ANOVA (Kruskal–Wallis with Dunn’s multiple comparisons post-test) using Prism5. Cross-comparisons were performed only if the overall *P* value of the ANOVA was <0.05.; error bars: s.d.

### Data Availability

The data that support the findings of this study and the custom code for the Delaunay triangulation application of Matlab software are available from the corresponding author upon reasonable request.

## Electronic supplementary material

Description of Additional Supplementary Files

Supplementary Movie 1

Supplementary Movie 2

Supplementary Movie 3

Supplementary Movie 4

Supplementary Movie 5

Supplementary Information
