# Supplementary material for: TBC1d24-ephrinB2 interaction regulates contact inhibition of locomotion in neural crest cell migration
Source: Nat Commun. 2018 Aug 28;9:3491. doi: 10.1038/s41467-018-05924-9 (PMC6113226; doi:10.1038/s41467-018-05924-9)
Supplement: Supplementary file 1 — Description of Additional Supplementary Files [file 41467_2018_5924_MOESM1_ESM.pdf]

Description of Additional Supplementary Files

File Name: Supplementary Movie 1
Description: Time-lapse movie of CNC cell migration. Time-lapse Images for in vitro migration of neural crest cell explants. MOs and RNAs were co-injected with the GFD into the D.1.2 blastomere at the 16 cell stage. Dissected CNC cells were incubated on fibronectin coated plates in MDM after brief dissociation in Ca^{2+}/Mg^{2+}- free MDM for 5 min. One frame per 1 min. The arrow head indicates the cell-cell contact. Panel 1 (from left, top): Control MO-injected CNC cells. Panel 2: TBC1d24 MO-injected CNC cells. Panel 3: TBC1d24 MO- and TBC1d24-WT-MOR RNA-injected CNC cells. Panel 4: TBC1d24 MO and TBC1d24-ΔD RNA-injected CNC cells. Panel 5: TBC1d24 MO and TBC1d24-ΔF RNA-injected CNC cells.

File Name: Supplementary Movie 2
Description: Time-lapse Images for sub-cellular localisation of GFP-Rab35 (green) and Clip-E-cadherin (red) depicted in Fig 5f. MOs and RNAs were co-injected with the GFP-Rab35 and Clip-E-cadherin into the D.1.2 blastomere at the 16-cell stage. Dissected CNC cells were incubated on fibronectin coated plates in MDM. One frame per 2 seconds. 1st row: Control MO-injected CNC cells. 2nd row: TBC1d24 MO-injected CNC cells. 3rd row: TBC1d24 MO and TBC1d24-WT-MOR RNA-injected CNC cells. 4th row: TBC1d24 MO and TBC1d24-ΔD RNA-injected CNC cells. 5th row: TBC1d24 MO and TBC1d24-ΔF RNA-injected CNC cells.

File Name: Supplementary Movie 3
Description: Time-lapse Images for sub-cellular localisation of GFP-Rab35 (green) and Clip-E-cadherin (red) depicted in Supplementary Fig. 3h. MOs and RNAs were co-injected with the GFP-Rab35 and Clip-E-cadherin into the D.1.2 blastomere at the 16-cell stage. Dissected CNC cells were incubated on fibronectin coated plates in MDM. One frame per 2 seconds. 1st row: Control MO-injected CNC cells. 2nd row: ephrinB2 MO-injected CNC cells. 3rd row: ephrinB2 MO and ephrinB2-WT-MOR RNA-injected CNC cells. 4th row: ephrinB2 MO and ephrinB2-Δ4 RNA-injected CNC cells.

File Name: Supplementary Movie 4
Description: Time-lapse Images for invasion assay depicted in Fig 6g. MOs were co-injected with the RFD into the D.1.2 blastomere at the 16-cell stage. Dissected CNC tissue (green) and PL tissue (red) were incubated together on fibronectin coated plates in MDM. One frame per 1 min. 1st row: Control CNC and Control PL. 2nd row: Control CNC and EphB4 MO-injected PL. Yellow indicates the overlapping region.

File Name: Supplementary Movie 5
Description: Time-lapse Images for chemoattraction assay depicted in Fig 6h. Dissected CNC tissue (injected with GFD) and PL tissue (injected with RFD) were incubated 400 μm apart on fibronectin coated plates in low Ca^{2+}/Mg^{2+} MDM with Control-Fc (3μg/ml) or clustered EphB4-Fc (3μg/ml). One frame per 10 min.
