# Supplementary material for: TBC1d24-ephrinB2 interaction regulates contact inhibition of locomotion in neural crest cell migration
Source: Nat Commun. 2018 Aug 28;9:3491. doi: 10.1038/s41467-018-05924-9 (PMC6113226; doi:10.1038/s41467-018-05924-9)
Supplement: Supplementary file 7 — Supplementary Information [file 41467_2018_5924_MOESM7_ESM.pdf]

TBC1d24-ephrinB2 interaction regulates contact inhibition of locomotion in neural crest cell migration

Yoon *et al.*

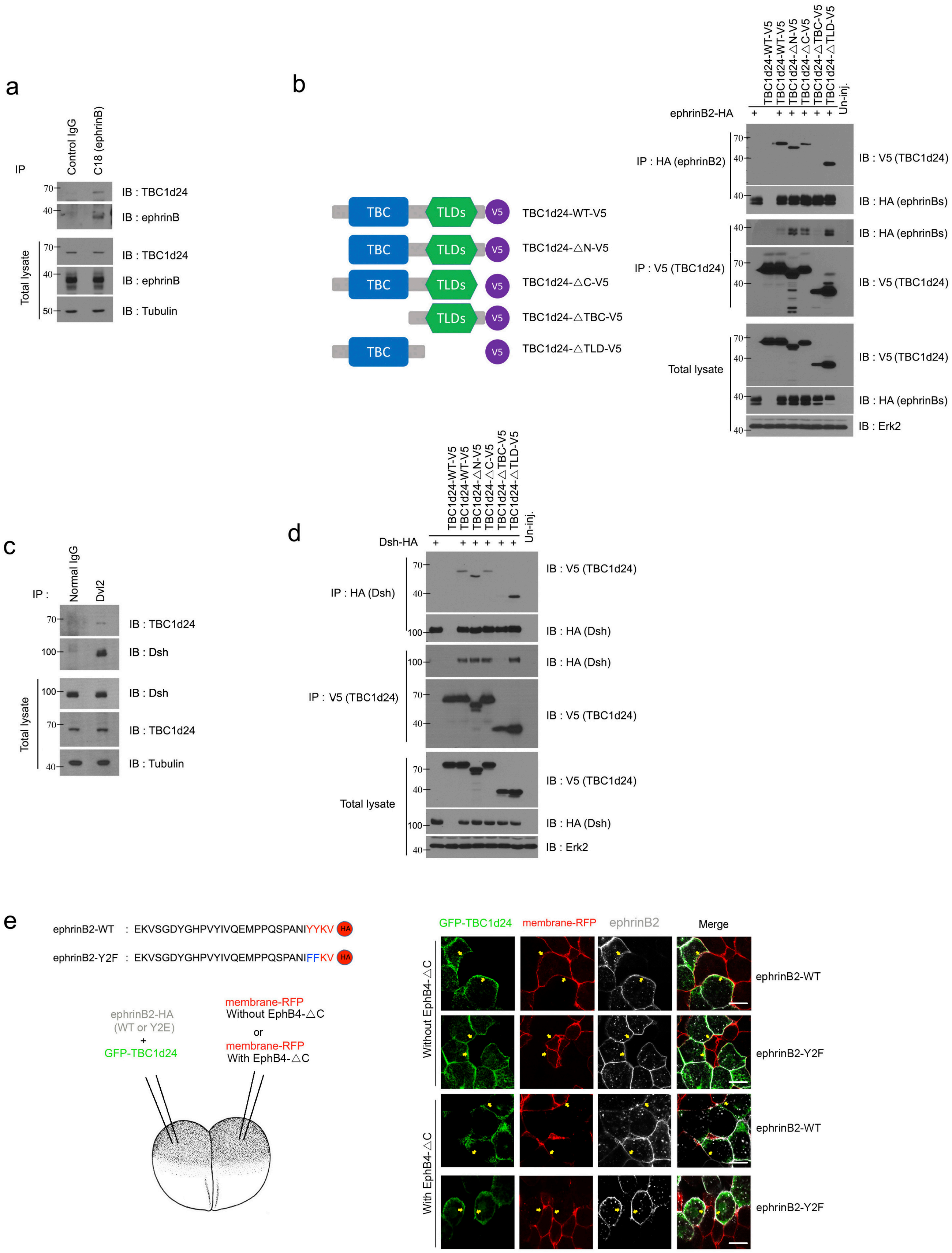

a
IP
Control IgG
C18 (ephrinB)
IB : TBC1d24
IB : ephrinB
Total lysate
IB : TBC1d24
IB : ephrinB
IB : Tubulin
70
40
70
40
50
b
TBC
TLDs
V5
TBC1d24-WT-V5
TBC1d24-△N-V5
TBC1d24-△C-V5
TBC1d24-△TBC-V5
TBC1d24-△TLD-V5
ephrinB2-HA
TBC1d24-WT-V5
TBC1d24-WT-V5
TBC1d24-△N-V5
TBC1d24-△C-V5
TBC1d24-△TBC-V5
TBC1d24-△TLD-V5
Un-inj.
IP : HA (ephrinB2)
IP : V5 (TBC1d24)
Total lysate
IB : V5 (TBC1d24)
IB : HA (ephrinBs)
IB : HA (ephrinBs)
IB : V5 (TBC1d24)
IB : V5 (TBC1d24)
IB : HA (ephrinBs)
IB : Erk2
c
IP :
Normal IgG
Dvl2
IB : TBC1d24
IB : Dsh
Total lysate
IB : Dsh
IB : TBC1d24
IB : Tubulin
70
100
100
70
40
d
Dsh-HA
TBC1d24-WT-V5
TBC1d24-WT-V5
TBC1d24-△N-V5
TBC1d24-△C-V5
TBC1d24-△TBC-V5
TBC1d24-△TLD-V5
Un-inj.
IP : HA (Dsh)
IP : V5 (TBC1d24)
Total lysate
IB : V5 (TBC1d24)
IB : HA (Dsh)
IB : HA (Dsh)
IB : V5 (TBC1d24)
IB : V5 (TBC1d24)
IB : HA (Dsh)
IB : Erk2
e
ephrinB2-WT : EKVSGDYGHPVYIVQEMPPQSPANIYYKV HA
ephrinB2-Y2F : EKVSGDYGHPVYIVQEMPPQSPANIFFKV HA
ephrinB2-HA
(WT or Y2E)
+
GFP-TBC1d24
membrane-RFP
Without EphB4-△C
or
membrane-RFP
With EphB4-△C
GFP-TBC1d24
membrane-RFP
ephrinB2
Merge
Without EphB4-△C
With EphB4-△C
ephrinB2-WT
ephrinB2-Y2F
ephrinB2-WT
ephrinB2-Y2F

Supplementary Figure 1. TBC1d24 interacts with ephrinB2 via Dsh. (a) Co-IP using control IgG or anti-ephrinB antibodies (C-18) shows that endogenous TBC1d24 binds to ephrinB2 in LS174T human colon cancer cells. (b) Illustration of the various TBC1d24 N and C-terminal deletion mutants. Co-IP using gastrula embryos injected with RNA encoding ephrinB2-HA and TBC1d24-V5 deletion mutants shows that the TBC domain of TBC1d24 is critical for an interaction with ephrinB2. (c) Co-IP using control IgG or anti-Dsh2 antibodies shows that endogenous TBC1d24 binds to Dsh2 in LS174T human colon cancer cells. (d) Co-IP using gastrula embryos injected with RNA encoding Dsh-HA and wildtype TBC1d24-V5 or domain deletion mutants of TBC1d24-V5 shows that the TBC domain is required for an interaction with Dsh. (e) Cartoon depicts the experimental design. IF in embryonic ectoderm shows that expression of wildtype ephrinB2 or ephrinB2-Y2F induces membrane localisation of GFP-tagged TBC1d24, which was unaffected at the site of cell-cell contact with control membrane-RFP expressing cells. However, membrane localisation of GFP-TBC1d24 in ephrinB2-WT expressing cells is dramatically reduced at the site of cell-cell contact with cells expressing both EphB4-ΔC and membrane-RFP. Co-expression of ephrinB2-Y2F maintains the membrane localisation of GFP-TBC1d24 at the site of cell-cell contact with EphB4-ΔC. EphrinB2 and/or TBC1d24 RNAs were injected as indicated. Animal caps were dissected at stage 10 and then immunostained for ephrinB2 (Alexa-647). The yellow arrow heads indicate cell-cell contact regions. Bar, 20 μm.

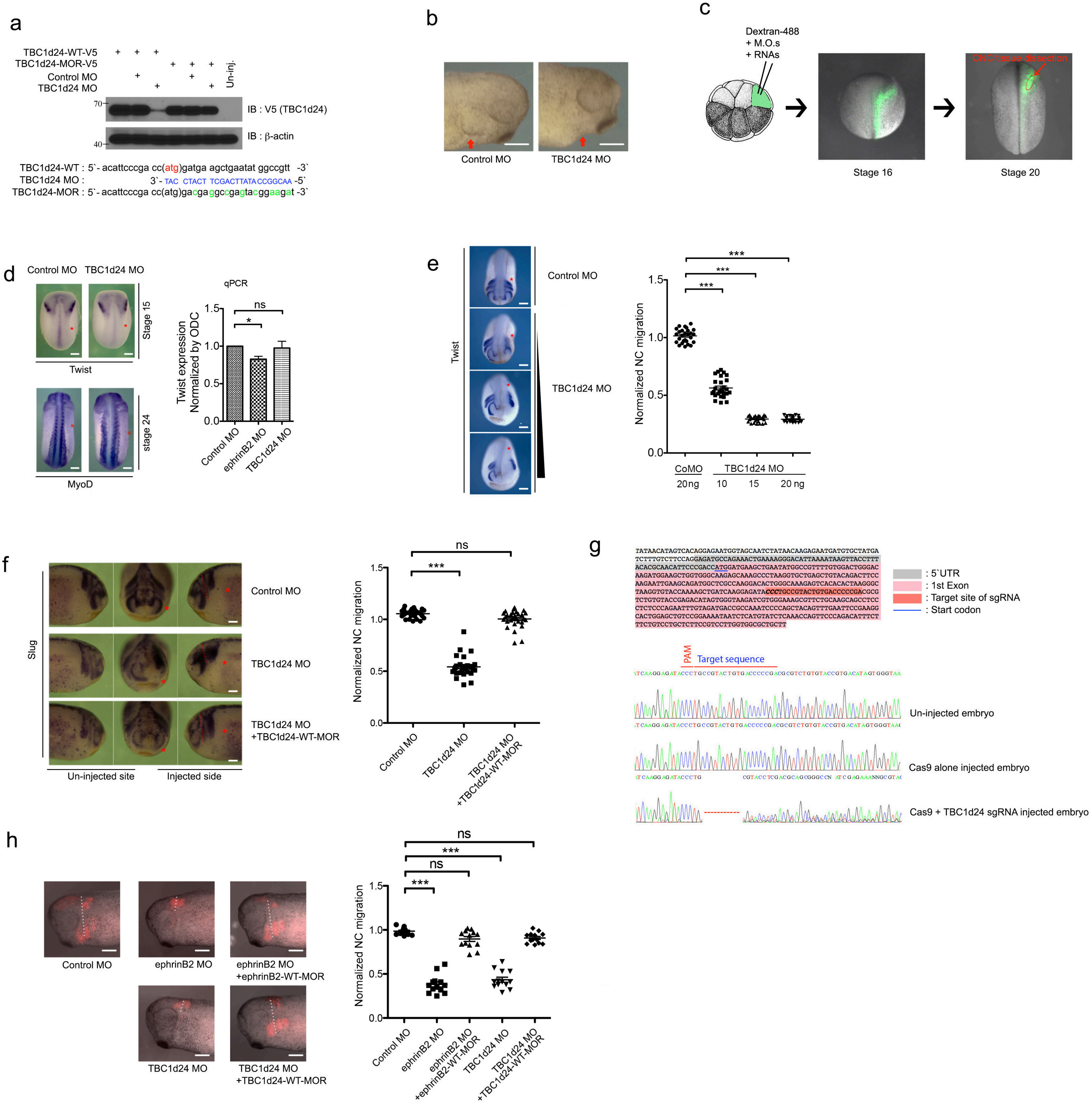

a
TBC1d24-WT-V5
TBC1d24-MOR-V5
Control MO
TBC1d24 MO
Un-inj.
IB : V5 (TBC1d24)
IB : β-actin
TBC1d24-WT : 5`- acattcccga cc(atg)gatga agctgaatat ggccgtt -3`
TBC1d24 MO : 3`- TAC CTACT TCGACTTATA CCGGCAA -5`
TBC1d24-MOR : 5`- acattcccga cc(atg)gacgaggccgagtacggaagat -3`
b
Control MO
TBC1d24 MO
c
Dextran-488
+ M.O.s
+ RNAs
CNC tissue dissection
Stage 16
Stage 20
d
Control MO
TBC1d24 MO
Stage 15
Twist
stage 24
MyoD
qPCR
Twist expression
Normalized by ODC
ns
Control MO
ephrinB2 MO
TBC1d24 MO
e
Twist
Control MO
TBC1d24 MO
Normalized NC migration
CoMO
20ng
TBC1d24 MO
10
15
20 ng
f
Slug
Control MO
TBC1d24 MO
TBC1d24 MO
+TBC1d24-WT-MOR
Un-injected site
Injected side
Normalized NC migration
ns
g
: 5`UTR
: 1st Exon
: Target site of sgRNA
: Start codon
PAM
Target sequence
Un-injected embryo
Cas9 alone injected embryo
Cas9 + TBC1d24 sgRNA injected embryo
h
Control MO
ephrinB2 MO
ephrinB2 MO
+ephrinB2-WT-MOR
TBC1d24 MO
TBC1d24 MO
+TBC1d24-WT-MOR
Normalized NC migration
ns

Supplementary Figure 2. TBC1d24 regulates CNC cell migration. (a) TBC1d24 MO effectively targets the translational start site. Western blot analysis from embryos injected with the indicated MOs and RNAs shows that the TBC1d24 MO efficiently blocks exogenous TBC1d24-WT expression whereas the expression of the MO-resistant mutant (TBC1d24-MOR) is not affected. Below the blot is a depiction of the wildtype TBC1d24 nucleotide sequence near the ATG start codon, the TBC1d24 MO, and the resistant RNA sequence. (b) Knockdown of TBC1d24 induces an aberrant pharyngeal pouch phenotype, indicative of a neural crest migration defect. 20 ng of Control MO or TBC1d24 MO was injected into 1-cell stage embryos that were culture to the tailbud stage. Bar, 200 µm. (c) Scheme for microinjection into the D.1.2 blastomere at the 16-cell stage. The D.1.2 blastomere is a major contributor to neural crest tissue and the lineage (green) can be traced at later stages. (d) WISH with *Twist* or *Myo-D* antisense probes show that TBC1d24 knockdown does not affect neural crest induction (*Twist*, at stage 15) or muscle patterning (Myo-D, at stage 24). Images are dorsal view with anterior at top. The red asterisk indicates the injected side. qPCR assay shows the relative *Twist* expression in ephrinB2 or TBC1d24 knockdown embryos (quantification with non-parametric ANOVA (Kruskal-Wallis test), $P = 0.0038$; Bar, 200 µm. (e) WISH with the *Twist* antisense probe shows TBC1d24 MO causes neural crest migration defects in a dose-dependent manner. TBC1d24 MO (10ng, 15ng or 20ng) was injected into one blastomere of 2-cell stage embryos, and cultured to stage 24; anterior-dorsal view, and red asterisk indicates the injected side (quantification with one-way ANOVA, $P < 0.0001$; Bar, 200 µm. (f) WISH with the Slug antisense probe shows TBC1d24 MO causes neural crest migration defects. Slug is used as another representative neural crest marker. Red asterisk indicates the injected side and red dotted line shows extent of CNC migration. The middle panels are an anterior view of embryos. (quantification with non-parametric ANOVA (Kruskal-Wallis test), $P < 0.0001$; Bar, 200 µm. (g) CRISPR/CAS9 targets the first exon of TBC1d24 gene. The DSP assay suggests that Cas9 and TBC1d24 sgRNA complex successfully create deletions at the target region in F0 embryos. (h) Neural crest transplant assay. Transferring the neural crest cells from ephrinB2 or TBC1d24 knockdown embryos (RFD) to a wildtype host causes neural crest cell migration defects that are rescued by expression of their MO resistance RNAs. White dotted line shows extent of CNC migration. Quantification with non-parametric ANOVA (Kruskal-Wallis test), $P < 0.0001$; Bar, 200 µm. All scatterplots represent mean ± s.e.m from three biological repeats; Dunn's multiple Comparison, *$P < 0.05$, **$P < 0.01$ and ***$P < 0.001$, NS: no statistical differences between groups.

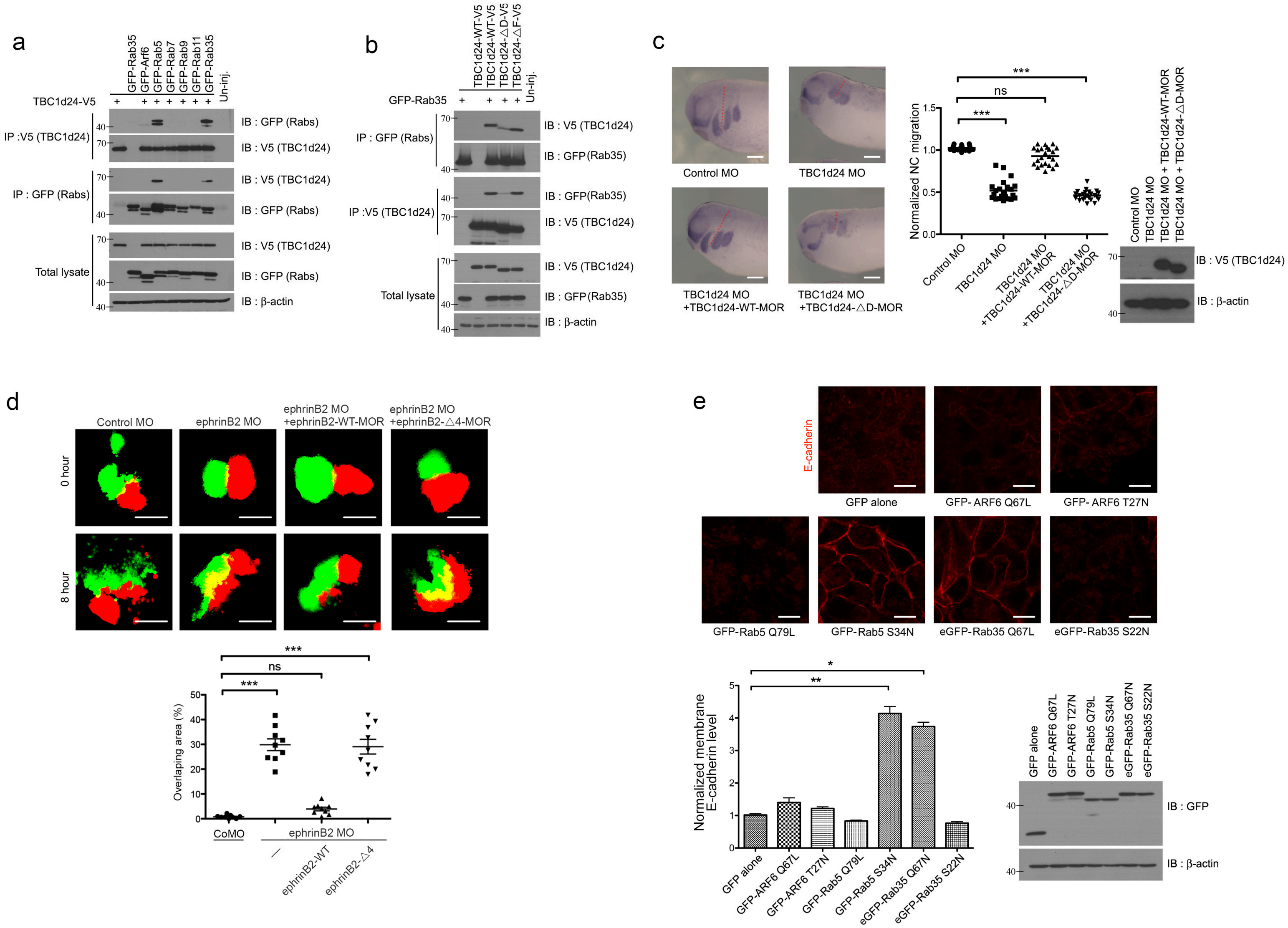

a
TBC1d24-V5
GFP-Rab35
GFP-Arf6
GFP-Rab5
GFP-Rab7
GFP-Rab9
GFP-Rab11
GFP-Rab35
Un-inj.
IP :V5 (TBC1d24)
IP : GFP (Rabs)
Total lysate
IB : GFP (Rabs)
IB : V5 (TBC1d24)
IB : β-actin
b
GFP-Rab35
TBC1d24-WT-V5
TBC1d24-△D-V5
TBC1d24-△F-V5
IP : GFP (Rabs)
IP :V5 (TBC1d24)
IB : V5 (TBC1d24)
IB : GFP(Rab35)
c
Control MO
TBC1d24 MO
TBC1d24 MO +TBC1d24-WT-MOR
TBC1d24 MO +TBC1d24-△D-MOR
Normalized NC migration
***
ns
TBC1d24 MO + TBC1d24-WT-MOR
TBC1d24 MO + TBC1d24-△D-MOR
d
Control MO
ephrinB2 MO
ephrinB2 MO +ephrinB2-WT-MOR
ephrinB2 MO +ephrinB2-△4-MOR
0 hour
8 hour
Overlaping area (%)
CoMO
ephrinB2-WT
ephrinB2-△4
e
E-cadherin
GFP alone
GFP- ARF6 Q67L
GFP- ARF6 T27N
GFP-Rab5 Q79L
GFP-Rab5 S34N
eGFP-Rab35 Q67L
eGFP-Rab35 S22N
Normalized membrane E-cadherin level
GFP-ARF6 Q67L
GFP-ARF6 T27N
eGFP-Rab35 Q67N
IB : GFP

f

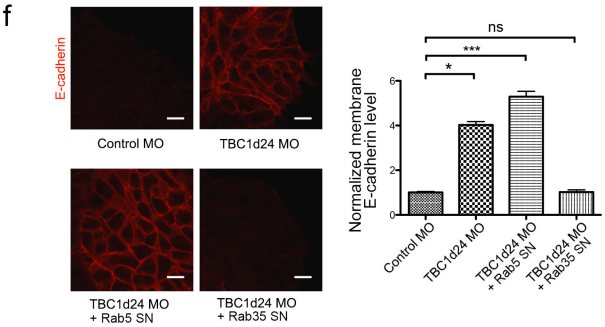
E-cadherin
Control MO
TBC1d24 MO
TBC1d24 MO
+ Rab5 SN
TBC1d24 MO
+ Rab35 SN
ns
***
*
Normalized membrane
E-cadherin level
6
4
2
0
Control MO
TBC1d24 MO
TBC1d24 MO
+ Rab5 SN
TBC1d24 MO
+ Rab35 SN

g

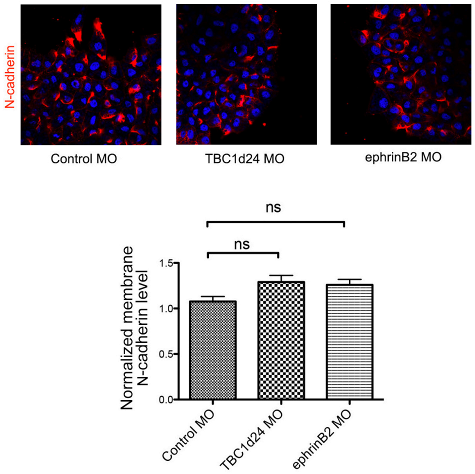
N-cadherin
Control MO
TBC1d24 MO
ephrinB2 MO
ns
ns
Normalized membrane
N-cadherin level
1.5
1.0
0.5
0.0
Control MO
TBC1d24 MO
ephrinB2 MO

h

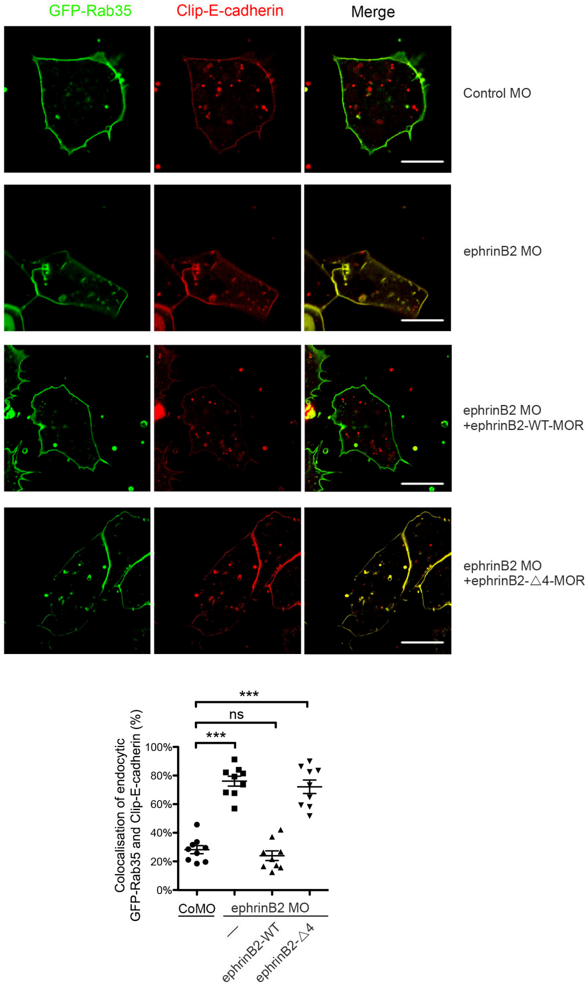
GFP-Rab35
Clip-E-cadherin
Merge
Control MO
ephrinB2 MO
ephrinB2 MO
+ephrinB2-WT-MOR
ephrinB2 MO
+ephrinB2-△4-MOR
***
ns
***
Colocalisation of endocytic
GFP-Rab35 and Clip-E-cadherin (%)
100%
80%
60%
40%
20%
0%
CoMO
ephrinB2 MO
—
ephrinB2-WT
ephrinB2-△4

Supplementary Figure 3. TBC1d24 regulates E-cadherin levels in CNC cells via Rab35. (a) Co-IP using gastrula embryos injected with RNA encoding ephrinB2-HA along with GFP-Rabs or GFP-Arf6 demonstrates that TBC1d24 interacts with Rab5 and Rab35 but only weakly with ARF6. (b) Co-IP using gastrula embryos injected with GFP-Rab35 and TBC1d24 deletion mutant RNAs shows that the D region in the TBC domain is critical for a Rab35 interaction. (c) WISH with the *Twist* antisense probe shows that knockdown of TBC1d24 induces neural crest cell migration defects that are rescued by expression of the wildtype TBC1d24. In contrast, the TBC1d24-ΔD mutant that cannot interact with Rab35 fails to rescue the TBC1d24 morphant defect. The red arrows indicate the migration differences between embryos. Quantification with non-parametric ANOVA (Kruskal-Wallis test), $P < 0.0001$; Bar, 200 μm. (d) Homotypic invasion assay. Explants excised from stage 20 embryos injected with the indicated MOs and RNAs were placed in juxtaposition. The invasion of explants labelled with GFD into RFD labelled explants was significantly increased by knockdown of ephrinB2 (ephrinB2 MO) in the GFD labelled explants as evidenced by the elevated overlap region (yellow). Expression of MO-resistant wildtype ephrinB2 (ephrinB2-WT-MOR) diminished the invasive behaviour. However, expression of the ephrinB2-Δ4 mutant (lacking the ability to interact with TBC1d24) fails to inhibit the invading ephrinB2-depleted explants. Quantification with one-way ANOVA, $P < 0.0001$; Bar, 200 μm. (e) IF using an E-cadherin Ab shows that expressing GFP fusion proteins of Rab5-S34N (dominant negative form) or Rab35-Q67L (constitutively active form) increases E-cadherin levels in CNC cells whereas ARF6 has no effect. Western analysis shows that these GFP-fused small GTPase mutants are expressed at similar levels. Quantification with non-parametric ANOVA (Kruskal-Wallis test), $P < 0.0001$; Bar, 20 μm. (f) IF in CNC explants using an E-cadherin Ab shows that knockdown of TBC1d24 (TBC1d24 MO) increases E-cadherin levels, and this elevation is blocked by expression of GFP-Rab35-SN Histogram depicts quantification of the relative E-cadherin levels. Quantification with non-parametric ANOVA (Kruskal-Wallis test), $P < 0.0001$; Bar, 20 μm. (g) IF using a N-cadherin antibody shows that knockdown of TBC1d24 (TBC1d24 MO) or ephrinB2 (ephrinB2 MO) does not affect the N-cadherin levels in neural crest explants. Histogram depicts quantification of the relative N-cadherin levels. Quantification with one-way ANOVA, $P < 0.0001$; Bar, 50 μm. (h) Rab35 and endocytic Clip-E-cadherin co-localise when ephrinB2 is impaired. Fluorescent microscopy shows that ephrinB2 knockdown (ephrinB2 MO) significantly increases the co-localisation of GFP-Rab35 and endocytic Clip- tagged E-cadherin in CNC cells, which is prevented by expressing wildtype ephrinB2 (ephrinB2-WT-MOR). However, expression of the ephrinB2-Δ4 mutant fails to reduce co-localisation of Rab35 and endocytic Clip-E-cadherin. Quantification with one-way ANOVA, $P < 0.0001$; Bar, 10 μm. All scatterplots represent mean ± s.e.m from three biological repeats; Dunn's multiple Comparison, $*P < 0.05$, $**P < 0.01$ and $***P < 0.001$, NS: no statistical differences between groups.

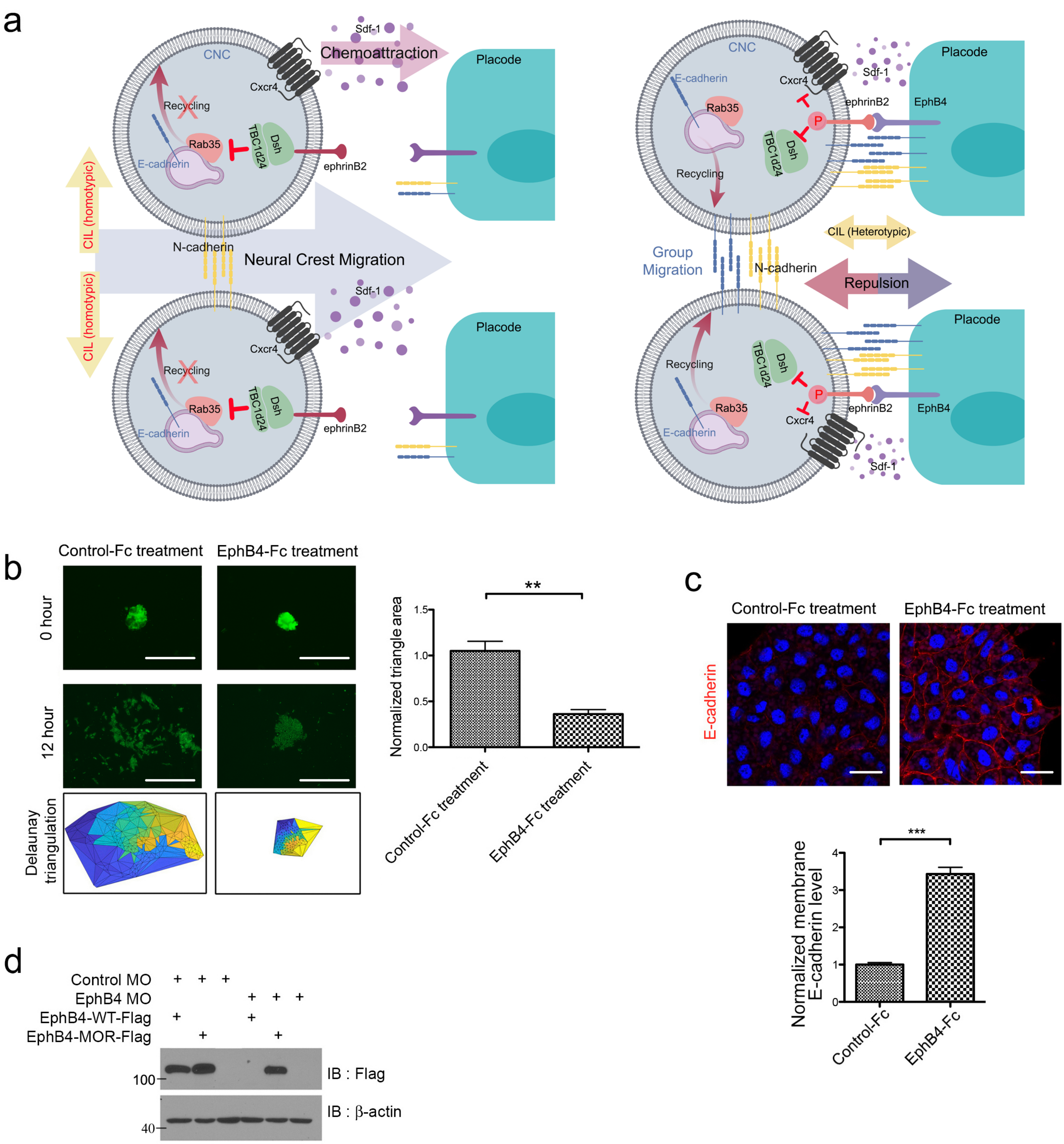
a
CNC
Sdf-1
Chemoattraction
Placode
Cxcr4
Recycling
Rab35
TBC1d24
Dsh
E-cadherin
ephrinB2
CIL (homotypic)
N-cadherin
Neural Crest Migration
EphB4
Group Migration
CIL (Heterotypic)
Repulsion
P
b
Control-Fc treatment
EphB4-Fc treatment
0 hour
12 hour
Delaunay triangulation
**
Normalized triangle area
1.5
1.0
0.5
0.0
Control-Fc treatment
EphB4-Fc treatment
c
Control-Fc treatment
EphB4-Fc treatment
E-cadherin
***
Normalized membrane E-cadherin level
4
3
2
1
0
Control-Fc
EphB4-Fc
d
Control MO
EphB4 MO
EphB4-WT-Flag
EphB4-MOR-Flag
100
40
IB : Flag
IB : β-actin

Supplementary Figure 4. EphrinB2/EphB4 signalling regulates neural crest migration. (a) Model indicates that ephrinB2 interacts with Dsh (via the DEP domain), which in turn interacts with TBC1d24 (via the TBC domain). TBC1d24 is recruited to the membrane and negatively regulates Rab35, resulting in diminished E-cadherin recycling to the membrane. Upon interacting with the cognate EphB4 receptor, ephrinB2 is tyrosine phosphorylated in the intracellular domain, causing Dsh to disengage from ephrinB2. This releases TBC1d24 from the membrane and thus localises it to the cytoplasm, allowing Rab35 to cycle E-cadherin to the membrane and block homotypic CIL. The binding of the Eph receptor to ephrinB2 also leads to reduced responsiveness of CNC cells to SDF-1-driven chemotaxis, but repulsive activity between ligand and receptor is initiated. (b) Cell dispersion assay. Neural crest tissues dissected from GFD-labeled embryos were incubated on fibronectin coated plates and treated with clustered Control-Fc or EphB4-Fc. Neural crest cells show a dispersed pattern in clustered Control-Fc after a 12-hour incubation. However, clustered EphB4-Fc treatment suppresses CNC cell dispersion. Delaunay triangulation analysis shows the triangle area between the neighboring cells significantly reduced by clustered EphB4-Fc treatment. Histogram quantifying average triangles area is shown in below panel**.** (Student's *t*-test, two-tailed). Bar, 400 μm. (c) Neural crest tissues were incubated on fibronectin coated plates and treated with clustered Control-Fc or EphB4-Fc for 3 hours. IF using the E-cadherin antibody shows that treatment with clustered EphB4-Fc increases E-cadherin levels in neural crest cells when compared with Control-Fc. Student's *t*-test, two-tailed). Bar, 20 μm. (d) Western blot analysis shows that EphB4 MO efficiently blocks EphB4-WT-Flag expression whereas the expression of EphB4-MOR-Flag is not affected by EphB4 MO. Histogram represents means ± s.e.m from three biological repeats; $^{**}P < 0.01$ and $^{***}P < 0.001$.

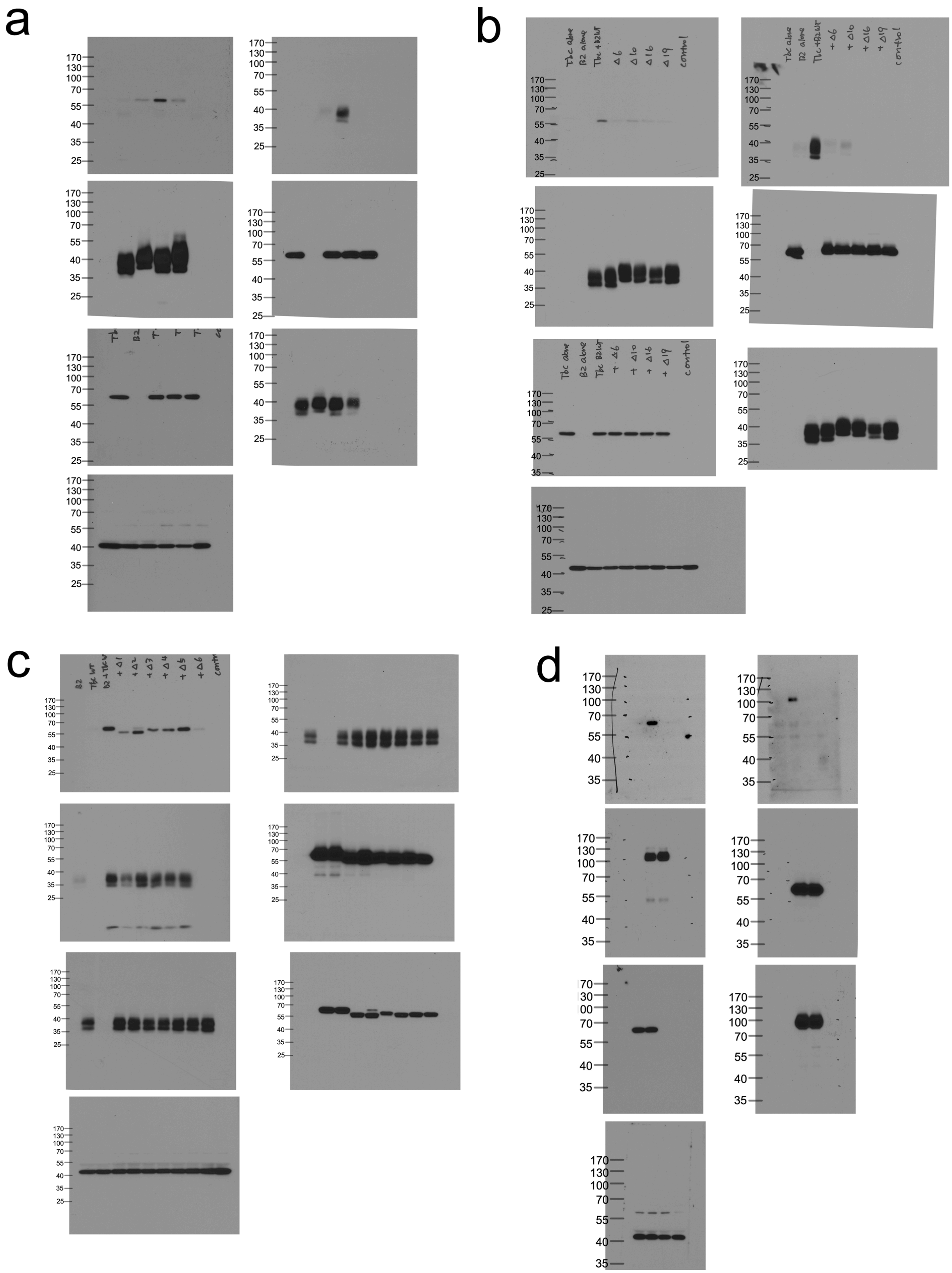
a
b
c
d
170
130
100
70
55
40
35
25
Tbc alone
B2 alone
Tbc + B2 WT
control

e

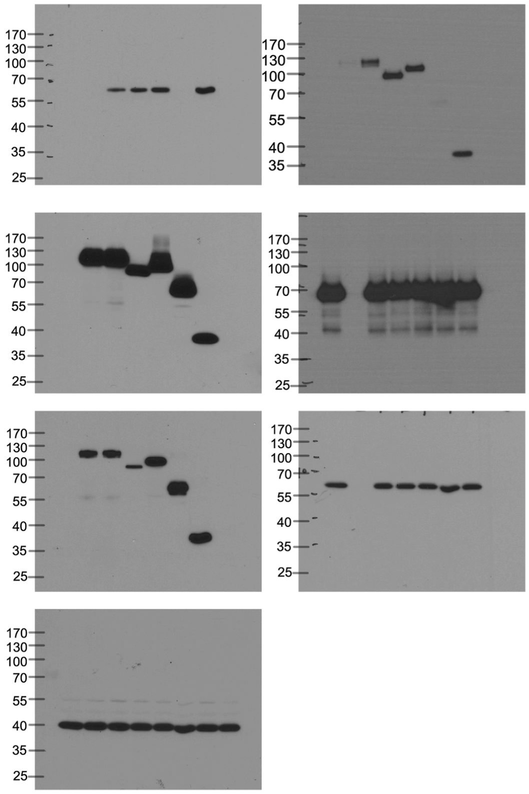
170
130
100
70
55
40
35
25

f

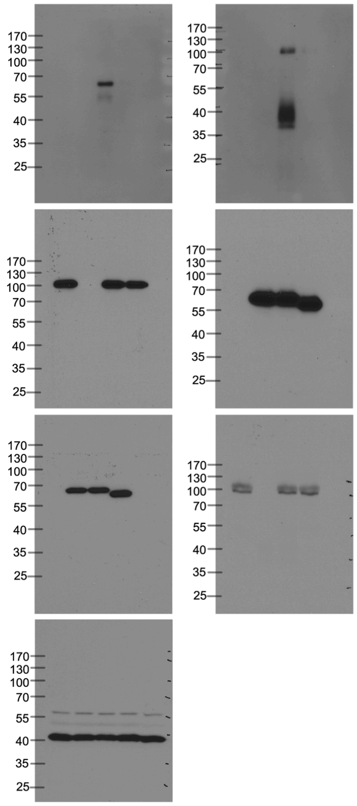
170
130
100
70
55
40
35
25

g

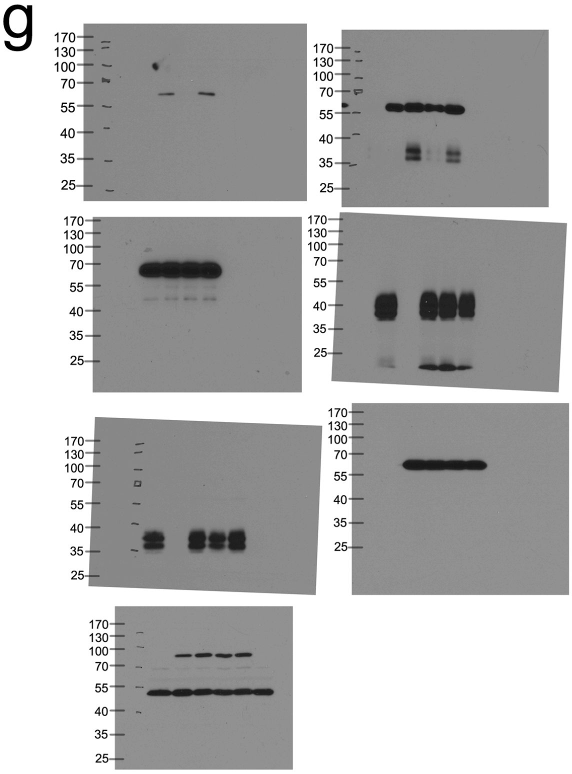
170
130
100
70
55
40
35
25

h

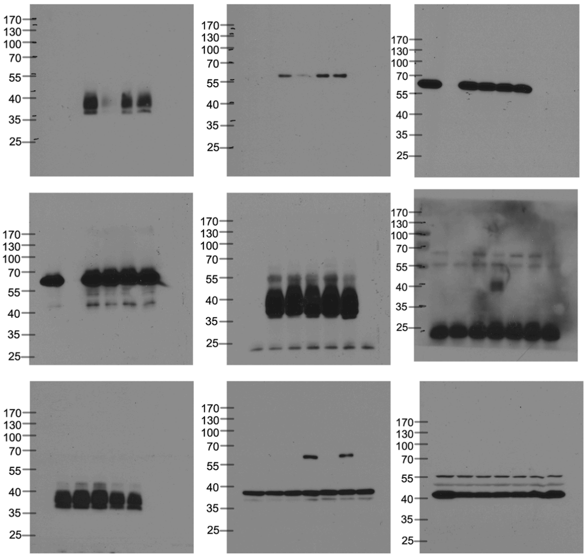
170
130
100
70
55
40
35
25

i

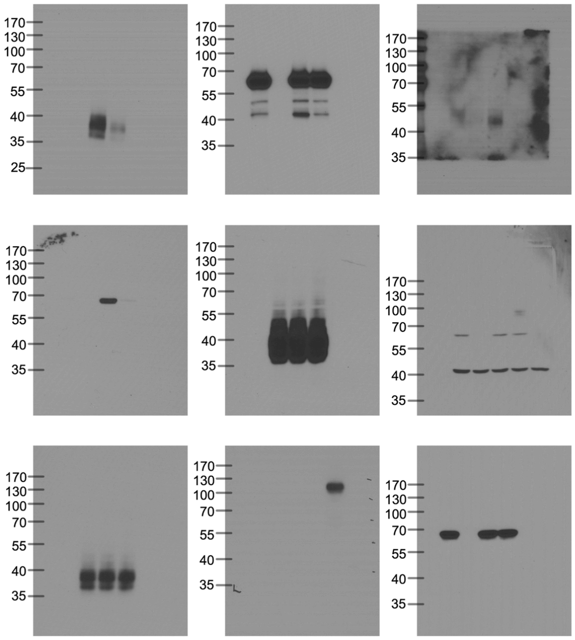

j

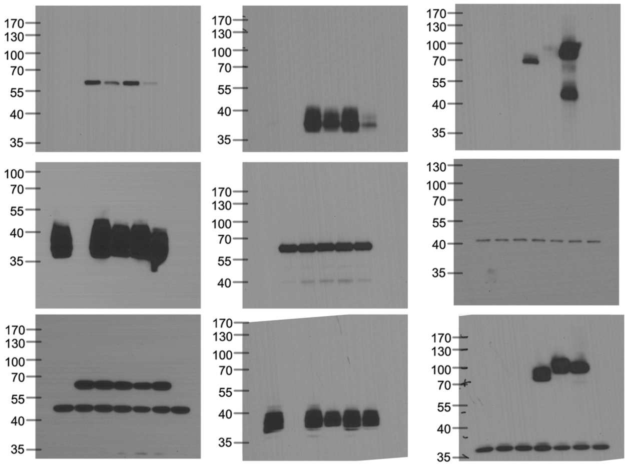

k

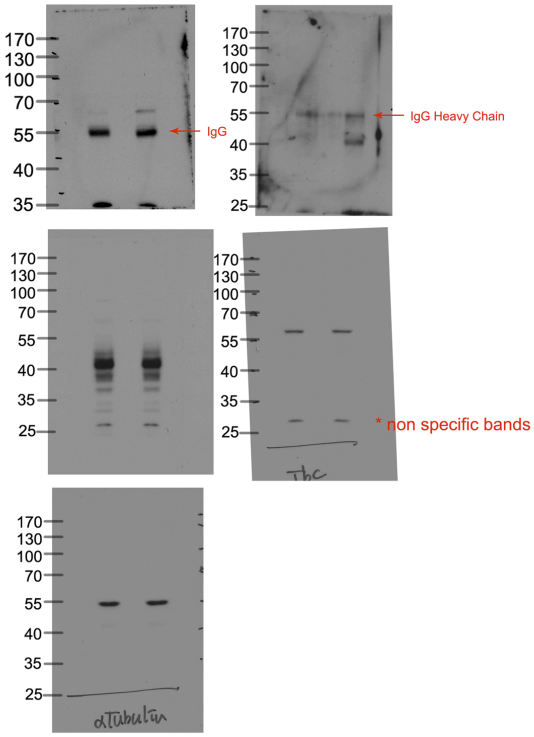

l

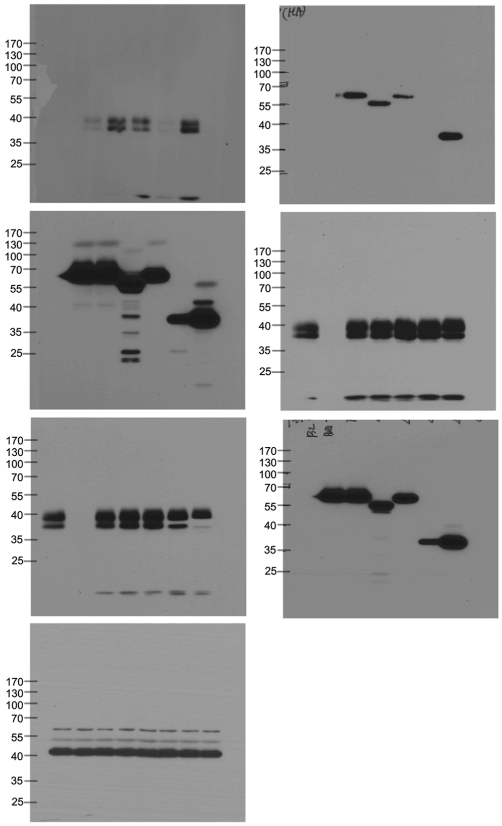

m

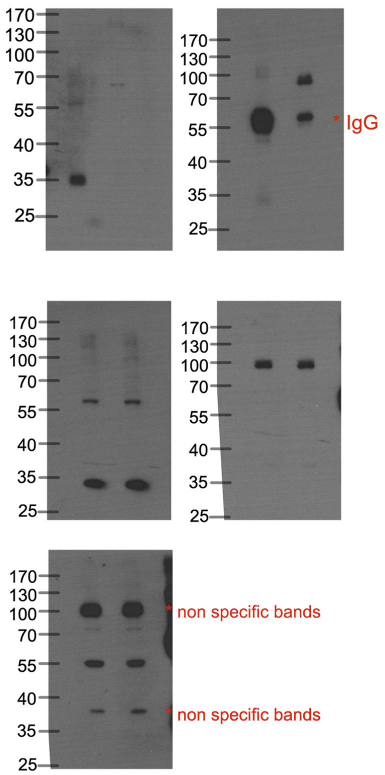
170
130
100
70
55
40
35
25
* IgG
* non specific bands
* non specific bands

n

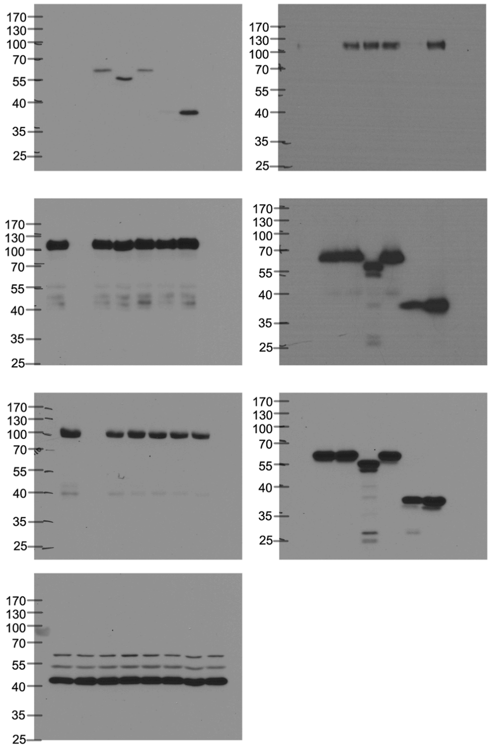
170
130
100
70
55
40
35
25

o

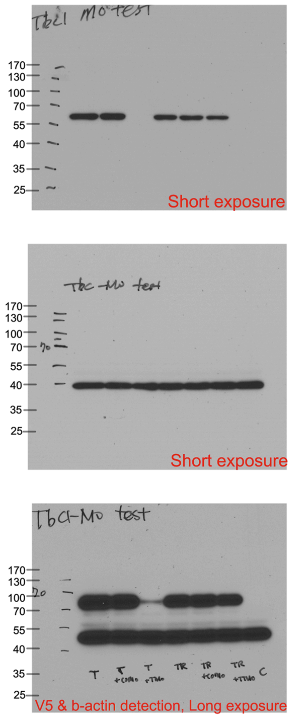
Short exposure
Short exposure
V5 & b-actin detection, Long exposure

p

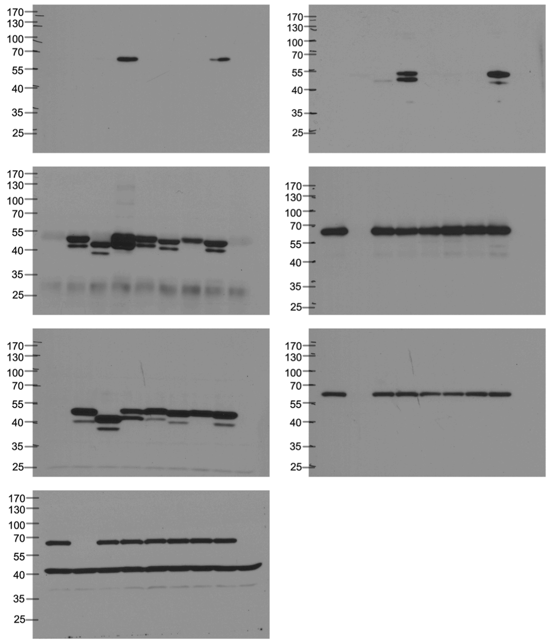
170
130
100
70
55
40
35
25

q

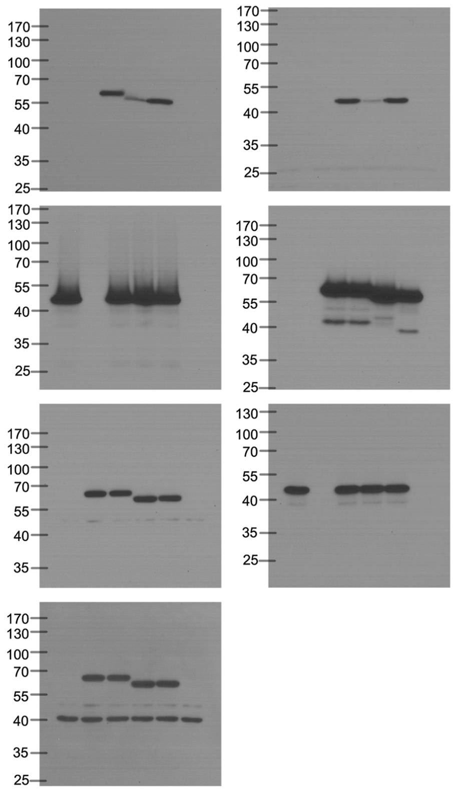

r

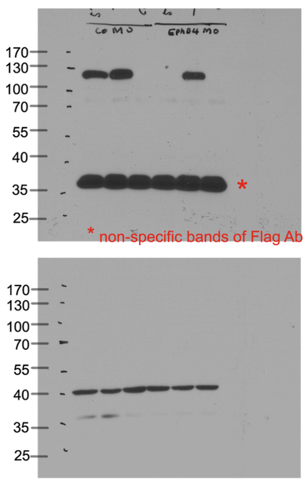

Supplementary Figure 5. Uncropped images. (a) Figure 1a, (b) Figure 1b, (c) Figure 1c, (d) Figure 2a, (e) Figure 2b, (f) Figure 2c, (g) Figure 2d, (h) Figure 3a, (i) Figure 3b, (j) Figure 6b, (k) Supplementary Figure 1a, (l) Supplementary Figure 1b, (m) Supplementary Figure 1c, (n) Supplementary Figure 1d, (o) Supplementary Figure 2a, (p) Supplementary Figure 3a, (q) Supplementary Figure 3b and (r) Supplementary Figure 4d.
